# Metformin inhibits proliferation of residing fibroadipogenic progenitor cells from failing human hearts

**DOI:** 10.1093/eschf/xvag001

**Published:** 2026-01-22

**Authors:** Anders Hostrup Larsen, Lin Lin, Andreas Buch Møller, Jean Farup, Lars Poulsen Tolbod, Tine Billeskov, Jonas Brorson, Elias Immanuel Ordell Sundelin, Steen Jakobsen, Helene Nørrelund, Hendrik Johannes Harms, Nils Henrik Hansson, Steen Bønløkke Pedersen, Lars Christian Gormsen, Yonglun Luo, Frank Vincenzo de Paoli, Jørgen Frøkiær, Henrik Wiggers, Niels Jessen

**Affiliations:** Steno Diabetes Center Aarhus, Aarhus University Hospital, Aarhus DK-8200, Denmark; Department of Cardiology, Aarhus University Hospital, Aarhus, Denmark; Department of Cardiology, Gødstrup Hospital, Herning, Denmark; Steno Diabetes Center Aarhus, Aarhus University Hospital, Aarhus DK-8200, Denmark; Department for Biomedicine, Aarhus University, Aarhus, Denmark; Steno Diabetes Center Aarhus, Aarhus University Hospital, Aarhus DK-8200, Denmark; Steno Diabetes Center Aarhus, Aarhus University Hospital, Aarhus DK-8200, Denmark; Department for Biomedicine, Aarhus University, Aarhus, Denmark; Department of Nuclear Medicine and PET Centre, Aarhus University Hospital, Aarhus, Denmark; Steno Diabetes Center Aarhus, Aarhus University Hospital, Aarhus DK-8200, Denmark; Steno Diabetes Center Aarhus, Aarhus University Hospital, Aarhus DK-8200, Denmark; Department of Nuclear Medicine and PET Centre, Aarhus University Hospital, Aarhus, Denmark; Steno Diabetes Center Aarhus, Aarhus University Hospital, Aarhus DK-8200, Denmark; Department of Endocrinology and Internal Medicine, Aarhus University Hospital, Aarhus, Denmark; Department of Nuclear Medicine and PET Centre, Aarhus University Hospital, Aarhus, Denmark; Department of Clinical Medicine, Aarhus University Hospital, Aarhus, Denmark; Department of Nuclear Medicine and PET Centre, Aarhus University Hospital, Aarhus, Denmark; Department of Cardiology, Aarhus University Hospital, Aarhus, Denmark; Steno Diabetes Center Aarhus, Aarhus University Hospital, Aarhus DK-8200, Denmark; Department of Nuclear Medicine and PET Centre, Aarhus University Hospital, Aarhus, Denmark; Steno Diabetes Center Aarhus, Aarhus University Hospital, Aarhus DK-8200, Denmark; Department for Biomedicine, Aarhus University, Aarhus, Denmark; Department for Biomedicine, Aarhus University, Aarhus, Denmark; Department of Cardiothoracic and Vascular Surgery, Aarhus University Hospital, Aarhus, Denmark; Department of Nuclear Medicine and PET Centre, Aarhus University Hospital, Aarhus, Denmark; Department of Clinical Medicine, Aarhus University Hospital, Aarhus, Denmark; Department of Cardiology, Aarhus University Hospital, Aarhus, Denmark; Steno Diabetes Center Aarhus, Aarhus University Hospital, Aarhus DK-8200, Denmark; Department for Biomedicine, Aarhus University, Aarhus, Denmark; Department of Clinical Pharmacology, Aarhus University Hospital, Aarhus, Denmark

**Keywords:** Fibrosis, Stem cells, Heart failure, Progenitor cells, FAPs, Metformin

## Abstract

**Introduction:**

Evidence from randomized trials indicates beneficial effects of metformin treatment in heart failure with reduced ejection fraction (HFrEF), but mechanisms of action remain elusive. We investigated myocardial metformin distribution *in vivo* in HFrEF patients and explored its effects on cardiac fibrogenic progenitor cells from human HFrEF hearts *in vitro*.

**Methods:**

We assessed myocardial metformin distribution and its dependency on myocardial viability in seven HFrEF patients (ejection fraction: 36 ± 8%; median age: 67 years) using ^11^C-metformin positron emission tomography (PET), ^15^O-H_2_O-PET, and exercise stress echocardiography. We characterized myocardial cellular composition by fluorescence-activated cell sorting and single-cell RNA sequencing (scRNA-seq) on mononuclear cells isolated from explanted left ventricles from four HFrEF patients and four control human hearts. A population of fibroadipogenic progenitor cells (FAPs) was identified and incubated after differentiation with metformin to test the effects on proliferation.

**Results:**

Myocardial ^11^C-metformin kinetics were best described by reversible two-tissue-compartment kinetics. Global myocardial metformin net influx rate was 0.012 ± 0.007 ml ml^−1^ min^−2^, and the myocardium-to-blood ratio was 1.24 (95% confidence intervals: 1.03–1.44; *P* < .001) after 90 min. Regional myocardial metformin net influx correlated inversely with myocardial viability (*r* = −0.65, *P* = .04). By scRNA-seq, we identified cardiac FAPs expressing *CD34* and *PDGFRA*, which transformed into extracellular matrix-forming myogenic cells upon activation. Incubation with metformin in clinically relevant doses (0.1 mM) inhibited FAP proliferation by 25%.

**Conclusion:**

Myocardial metformin uptake in HFrEF is marginal and confined to less viable and fibrotic regions. Cardiac FAPs are resident in human HFrEF myocardium and exhibit fibrogenic potential. Metformin inhibits FAP activation and proliferation at clinically relevant concentrations. These findings suggest that metformin may attenuate adverse left ventricular remodelling by targeting cardiac FAPs.

**Clinical trial registration:**

https://clinicaltrials.gov. Unique identifier: NCT03122769.

## Graphical Abstract

**Graphical Abstract:**
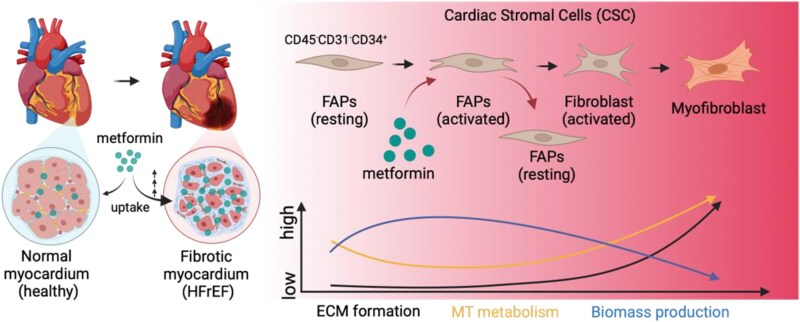

## Introduction

Metformin is a widely used antidiabetic drug with a well-established safety profile and low cost.^1^ In addition to lowering blood-glucose, observational studies suggest that metformin exerts pleiotropic cardioprotective effects in patients with Type 2 diabetes.^2–4^ Beyond diabetes, randomized clinical trials have shown that metformin improves myocardial efficiency and reduces myocardial oxygen consumption in patients with heart failure with reduced ejection fraction (HFrEF),^5^ and reverses left ventricular remodelling in non-diabetic patients with coronary artery disease.^6^ A large cardiovascular outcomes trial in HFrEF patients is currently ongoing.^7^ Together, these findings highlight the promise of repurposing metformin as a therapy for cardiovascular disease.^8^

Progressive myocardial fibrosis^9,10^ and adverse chamber remodelling impeding mechanical function are main characteristics of heart failure.^11^ These fibrotic changes are largely caused by non-muscle cells within the myocardium.^12^ Targeting these cells to inhibit or reverse of fibrosis is therefore considered a treatment strategy, already incorporated to some degree in standard HFrEF therapy with prognostic benefit.^11^ Although the mechanisms underlying the cardioprotective effects of metformin remain incompletely understood,^2^ preclinical rodent studies show that metformin attenuates myocardial fibrosis in HFrEF models,^13–17^ and observational clinical data from diabetes patients show reduced lipid accumulation in transplanted human hearts in patients treated with metformin compared with other antidiabetic drugs.^9^ Consistent with these observations, metformin has been shown to attenuate adipogenesis in skeletal muscle-resident progenitor cells by modulating intracellular signalling pathways.^18,19^ Hence, the purported beneficial cardiac effects of metformin may thus be mediated through effects on non-muscle cells, but this remains unexplored in human myocardial tissue.

Cellular uptake of metformin is a prerequisite for potential direct pharmacological actions, since metformin does not bind to extracellular receptors.^20^ Uptake depends on organic cation transporters (OCTs),^21^ and cardiac expression of the key transporters OCT1 and OCT3 is preserved in HFrEF irrespective of HFrEF aetiology.^22,23^ Although non-invasive biodistribution studies in healthy subjects have not shown appreciable cardiac uptake of metformin,^24^ it remains unexplored whether specific non-muscle myocardial cell types that emerge during HFrEF may facilitate metformin transport into the myocardium.

To address the hypothesis that metformin directly targets specific cells in the diseased myocardium, we conducted a clinical trial (NCT03122769) using *in vivo*  ^11^C-metformin positron emission tomography (PET) to investigate metformin distribution in HFrEF patients. In parallel, we analysed the cellular composition of explanted human left ventricles (four controls and four HFrEF patients) by single-cell RNA sequencing (scRNA-seq), and examined the effects of metformin on an identified population of cardiac fibroadipogenic progenitors (FAPs, *CD34*+, and *PDGFRA+*) to address the hypothesis of antifibrotic myocardial effects of metformin.

## Methods

Detailed descriptions of materials, methods, and protocols are provided in *Table 1* and the Supplementary material. In brief, this study contains data from two complementary investigations of HFrEF patients. First, we investigated the myocardial biodistribution of metformin *in vivo* in seven HFrEF patients using ^15^O-H_2_O PET and ^11^C-metformin PET. Second, we performed scRNA-seq (10× Genomics) of mononuclear cells isolated from the left ventricle of four explanted hearts from HFrEF patients undergoing orthotopic heart transplantations at Aarhus University Hospital, Denmark. Single-cell RNA sequencing data from control human heart (without HFrEF, hereafter referred as control) were collected from the Human Cell Atlas Data Coordination Platform with accession number: ERP123138. *In vitro* assays were performed to characterize and validate the response of non-cardiomyocyte cells residing in the human heart to metformin.

**Table 1:** Key resources table

Reagent or resource	Source	Identifier
Antibodies and probes		
Anti PLIN-1 (Rb)	Cell Signalling Technology	Cat. no. 9349
Anti COL-1 (Ms)	Sigma Aldrich	Cat. no. C2456
Anti CD31	Miltenyi	Cat. no. 130-110-673
Anti CD45	Miltenyi	Cat. no. 130-114-123
Anti CD34	Miltenyi	Cat. no. 555824
Anti Rb Alexa647	Thermo Fisher	Cat. no. A-21245
Anti Ms Alexa488	Thermo Fisher	Cat. no. A-11001
Actin Green probe	Thermo Fisher	Cat. no. R37110
Primers		
COL1A1 (112 bp)		
Forward	TGCGATGACGTGATCTGTGACG	
Reverse	TTTCTTGGTCGGTGGGTGACTCTG	
COL6A1 (72 bp)		
Forward	ATCAGCCAGACCATCGACACCATC	
Reverse	TTCGAAGGAGCAGCACACTTGC	
β-2 microglobulin (111 bp)		
Forward	GAGGCTATCCAGCGTACTCC	
Reverse	AATGTCGGATGGATGAAACCC	
Assays and ingredients		
Adipogenic media	Miltenyi	Cat. no. 130-091-677
Bioamf media	Clini Sciences	Cat. no. 01-194-1A
High glucose Dulbecco's Modified Eagle Medium (DMEM)	Thermo Fisher	Cat. no. 11965092
FBS	Thermo Fisher	Cat. no. 16000044
TGFβ1	Merck	Cat. no. T7039
EDU click it 488	Thermo Fisher	Cat. no. c10637
TUNEL click it 647	Thermo Fisher	Cat. no. c10247
Chromium Single Cell 3 GEM kit	10× Genomics	Cat. no. 1000075
Chromium Chip B Single Cell Kit	10× Genomics	Cat. no. 1000073
Compensation beads	Thermo Fisher	Cat. no. 01-2222-41
HiSeq3000/4000 PE Cluster Kit	Illumina Denmark ApS	Cat. no. PE-410-1001
HiSeq 3000/4000 SBS Kit	Illumina Denmark ApS	Cat. no. FC-410-1003
Metformin	Sigma Aldrich	Cat. no. D150959
Deposited data		
Single cell RNA sequencing	GEO (access number GAS50000000125)	
Software and algorithms		
Sigma Plot 14.0	Systat Software Inc.	http://www.sigmaplot.co.uk
R	Open source	http://www.r-project.org
aQuant	aQuant Research	http://medtracepharma.com/aquant
STATA 14.2, Stata	Stata Nordic	http://www.statanordic.com
EVOS M7000 Imaging	Thermo Fisher	http://www.thermofisher.com
FlowJo	FlowJo	http://www.flowjo.com

### Patient population

#### Patients included for ^11^C-metformin biodistribution analysis

Seven non-diabetic HFrEF patients with left ventricular ejection fraction <45%, New York Heart Association function Class I–III, and relatively preserved renal function (eGFR > 30 ml min^−1^ 1.73 m^−2^) were included. Exclusion criteria were metformin contraindications. Patients were recruited from the outpatient clinic, Department of Cardiology, Aarhus University Hospital, Denmark. Following an initial screening visit to evaluate in- and exclusion criteria, patients were examined by ^15^O-H_2_O-PET and subsequent ^11^C-metformin PET performed during a single visit. All patients provided written consent before inclusion. The study was conducted in accordance with the principles of the Helsinki Declaration and International Conference on Harmonization of Technical Requirements for Registration of Pharmaceuticals for Human Use-Good Clinical Practice guidelines. Approvals were obtained from the local scientific ethical committee in the Central Denmark Region, from the Danish Data Protection Agency, and from the Danish Medicines Agency. The trial is registered at ClinicalTrials.gov, NCT03122769. Patient characteristics are presented in Supplementary Table S1. The patients all had HFrEF due to ischaemic heart disease: five patients with previous inferior wall myocardial infarction and two patients with previous anterior wall myocardial infarction. Mean administered ^11^C-metformin activity was 141.8 ± 39 MBq (range, 75.9–195 MBq). Two patients experienced transient dizziness after injection that ceased within 10 s, and no other side effects were observed.

#### Heart transplant recipients

We collected human heart tissue from the left ventricle of explanted hearts from four HFrEF patients undergoing orthotopic heart transplantation at Aarhus University Hospital, Denmark. The patients were anonymized, and the explanted hearts were provided without personally identifiable information. All patients were male adults with end-stage HFrEF due to dilated cardiomyopathy (DCM) of which one patient had a history of at least one family member also diagnosed with DCM (familial DCM).

### Statistics

Normally distributed variables are presented as mean ± standard deviation. Non-normally distributed data are presented as median (interquartile range) or as individual data points and medians. Categorical variables are presented as absolute values and percentages. Comparisons of means were performed using Wilcoxon rank-sum or with repeated measures analysis of variance when appropriate. Area under the curve (AUC) was calculated using the trapezoidal rule. Linear regression models were used to compare continuous variables, and we used robust standard errors to account for clustering due to within-subject myocardial segmental dependency. Predicted values and residuals were used for model check. A two-tailed value of *P* < .05 was considered statistically significant. Standard statistical software packages were used (STATA/IC 14.2, StataCorp LP, College Station, TX, USA, and SigmaPlot, Systat Software Inc., UK).

## Results

### Myocardial uptake of metformin is marginal in heart failure patients

**Figure 1:**
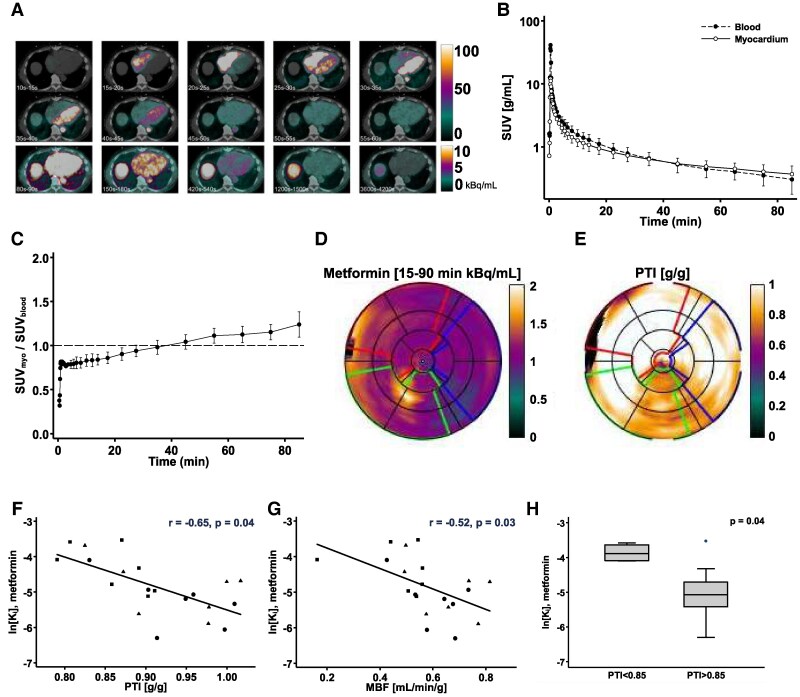
Biodistribution of ^11^C-metformin in the myocardium of heart failure patients with reduced ejection fraction. (*A*) Representative transaxial positron emission tomography images co-registered with low dose computed tomography. Positron emission tomography images are average ^11^C-metformin activity in the myocardium and the cranial part of the right hepatic lobe at specific time intervals. Activity is expressed as kBq/ml. (*B*) Time activity curves of ^11^C-metformin in blood (black) and myocardium (white) expressed as mean standardized uptake values on log scale. (*C*) Mean myocardium-to-blood ratio of ^11^C-metformin concentration at each time point. (*D*) Regional myocardial ^11^C-metformin activity (weighted average of 15–90 min) in vessel regions corresponding to septal (red), inferior (green), and lateral (blue) left ventricular myocardial wall regions. (*E*) Regional myocardial viability by ^15^O-H_2_O positron emission tomography-derived perfusable tissue index in septal (red), inferior (green), and lateral (blue) left ventricular myocardial wall regions of the left ventricle. (*F* and *G*) Scatter plots with regression lines showing the relation between metformin influx constant (*K_i_*) on log scale and perfusable tissue index (*F*) and myocardial blood flow (*G*) in septal (dots), inferior (squares), and lateral (triangles) left ventricular myocardial wall regions. (*H*) Box plot showing metformin influx constant (*K_i_*) on log scale stratified into viability, according to perfusable tissue index (*P < .05)

To investigate myocardial metformin uptake in HFrEF patients, we determined cardiac biodistribution by ^11^C-metformin PET. Global myocardial ^11^C-metformin activity is depicted in *Figure 1A* and shows marginal visual uptake. Both blood and myocardial activity peak shortly after injection, reaching a myocardial maximal standardized uptake value (SUV_max_) of ∼12 g ml^−1^ after 30 s with a subsequent rapid biphasic decline (*Figure 1B*). Myocardial ^11^C-metformin kinetics are best described by a two-tissue-compartment model with reversible binding, producing the lowest Akaike scores. The model yields low global myocardial metformin net influx of 0.012 ± 0.007 ml ml^−1^ min^−2^ and low volume of distribution (*V*_d_) of 0.63 ± 0.05 ml ml^−1^. Throughout the 90 min scan, AUC does not differ between blood and myocardium (AUC_blood_: 84.4 ± 23.4 vs AUC_myocardium_: 68.9 ± 18.0 g min ml^−1^; *P* = .19). The myocardium-to-blood ratio peaks at 1.24 (95% confidence interval, 1.03–1.44; *P* < .001) after 90 min (*Figure 1C*), indicating a marginal, if any, global myocardial uptake of metformin.

### Metformin uptake is confined to less viable and fibrotic regions of the myocardium in heart failure patients

To examine whether metformin uptake is higher in fibrotic regions of the heart, we compared regional left ventricular metformin influx with the ^15^O-H_2_O PET-derived perfusable tissue index (PTI), i.e. a marker of myocardial viability (*Figure 1D* and *E*). Linear regression analysis reveals a significant inverse correlation between regional myocardial metformin net influx and PET-derived PTI (*Figure 1F*) and myocardial blood flow (*Figure 1G*). When dichotomized by viability with a validated PTI cut-off value of 0.85, regions with reduced viability have significantly higher metformin net influx rates than viable regions (*Figure 1H*). Congruently, echocardiographic strain analysis reveals a significant correlation between regional myocardial metformin net influx and peak exercise segmental longitudinal strain (*r* = −0.18, *P* = .02). Thus, distribution of metformin is higher in less viable and fibrotic regions of the failing human heart.

### Single-cell transcriptome profiling of the failing myocardium

**Figure 2:**
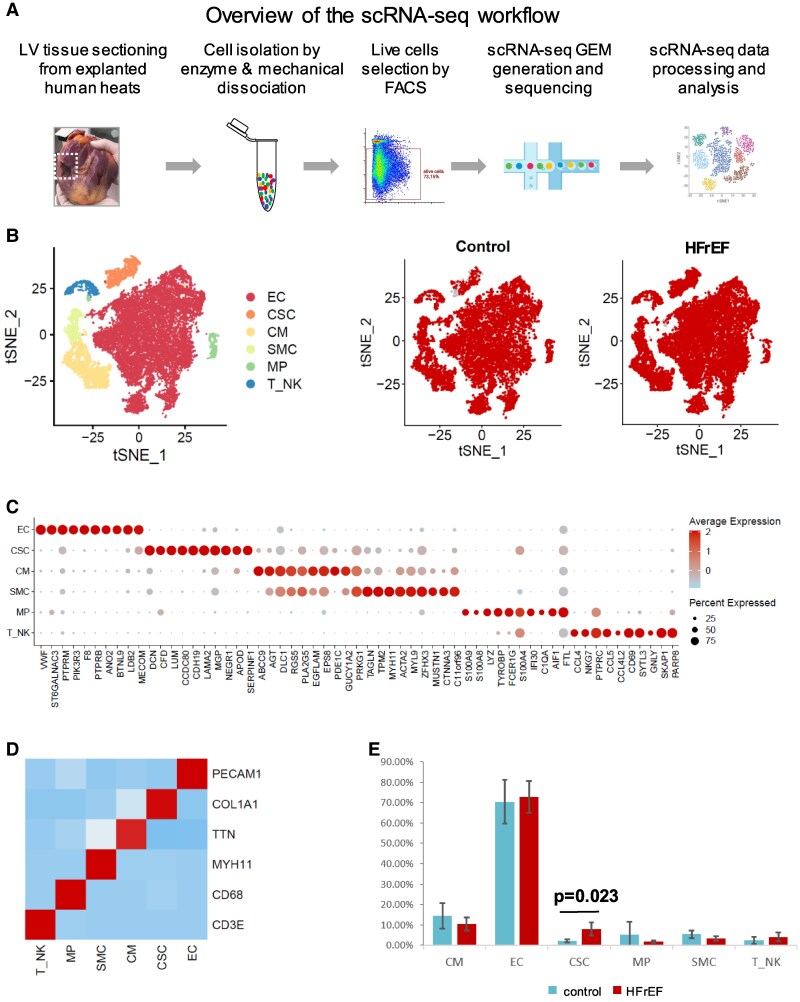
Single cell gene expression profiling of mononuclear cells isolated from the human myocardium. (*A*) Schematic of the workflow used to isolate mononuclear cells from the left ventricle of human hearts from heart transplantation recipients for single cell gene expression profiling. (*B*) Major cardiac cell populations identified after unsupervised clustering. Each point depicts a single cell coloured according to cluster designation. Spectral t-distributed stochastic neighbour embedding (tSNE) was used to reduce the data to two dimensions for visualization. EC, endothelial cells; CSC, cardiac stromal cells; CM, cardiomyocytes; SMC, smooth muscle cells; MP, macrophages; T_NK, T and NK cells. (*C*) Dot plot of 10 marker genes for each major cell cluster. Individual dots are sized to reflect the proportion of each type expressing the marker gene and coloured to reflect the mean expression of each marker gene across all cells. (*D*) Established cell type markers strongly and specifically associate with major cell types. The colour intensity in individual squares of the heatmap is the mean expression of each gene marked along the *y*-axis scaled according to the major cell types depicted at the *x*-axis (blue: lowest; red: highest). (*E*) Absolute and relative distribution of major cell types among Patient 1–5 and controls

To unravel the cardiac cell population in HFrEF, we analysed mononuclear cells from the left ventricle of explanted hearts from four HFrEF patients (*n* = 4) using scRNA-seq (Supplementary Figure S1A, *Figure 2A*) and compared with cells from non-HFrEF controls (hereafter referred as control). For scRNA-seq of HFrEF samples, we enriched live cells (propidium iodide stained negative) by fluorescence-activated cell sorting (FACS) and generated single-cell barcoded gene expression profiles using the 10× Genomics platform. High-quality scRNA-seq data were obtained, with an average of 41 000 reads per cell and a saturation rate of 68% (Supplementary Table S2). After filtering low quality cells and doublets, we obtained single-cell transcriptional profiles of 3500–5000 cells from each donor (Supplementary Figure S1C).

We next characterized the cardiac cell types based on single-cell transcriptome. After batch-correction, dimensional reduction, and unsupervised cell clustering based on highly variable genes (cf. Methods), we identified six major cell types (*Figure 2B*). Each cluster of cells exhibited strong and specific expression of canonical signature genes (*Figure 2C*). The top 50 marker genes enriched in each cell cluster were used for cell type annotation and were provided in Supplementary Table S3 (top gene transcripts with adjusted *P* < .05). To functionally annotate the cell type in each cluster, we examined the expression of canonical marker genes for human cardiac cells and combined with gene ontology (GO) enrichment analysis of the top markers. Using this strategy, the six major cardiac cell types captured by scRNA-seq, including endothelial cells (*PCAM1*), cardiac stromal cells (CSCs) (*COL1A1*, hereafter referred as CSCs), macrophages (*CD68*), T/NK cells (*CD3E*), cardiomyocytes (*TNN*), and smooth muscle cells (*MYH11*) (*Figure 2D*). Notably, although all six major cell types were found in both control and HFrEF hearts, there were significantly higher fraction of CSCs in HFrEF patients compared with non-HFrEF controls (*P* = .023, *Figure 2E*). Furthermore, to facilitate the exploration of all protein-coding gene expression levels in all the six cardiac cell types in control and HFrEF samples at the single-cell levels, we generated an interactive data visualization database (https://dreamapp.biomed.au.dk/heart_failure/) using the ShinyCell.^25^

### Activation of fibroadipogenic progenitors links to heart failure

**Figure 3:**
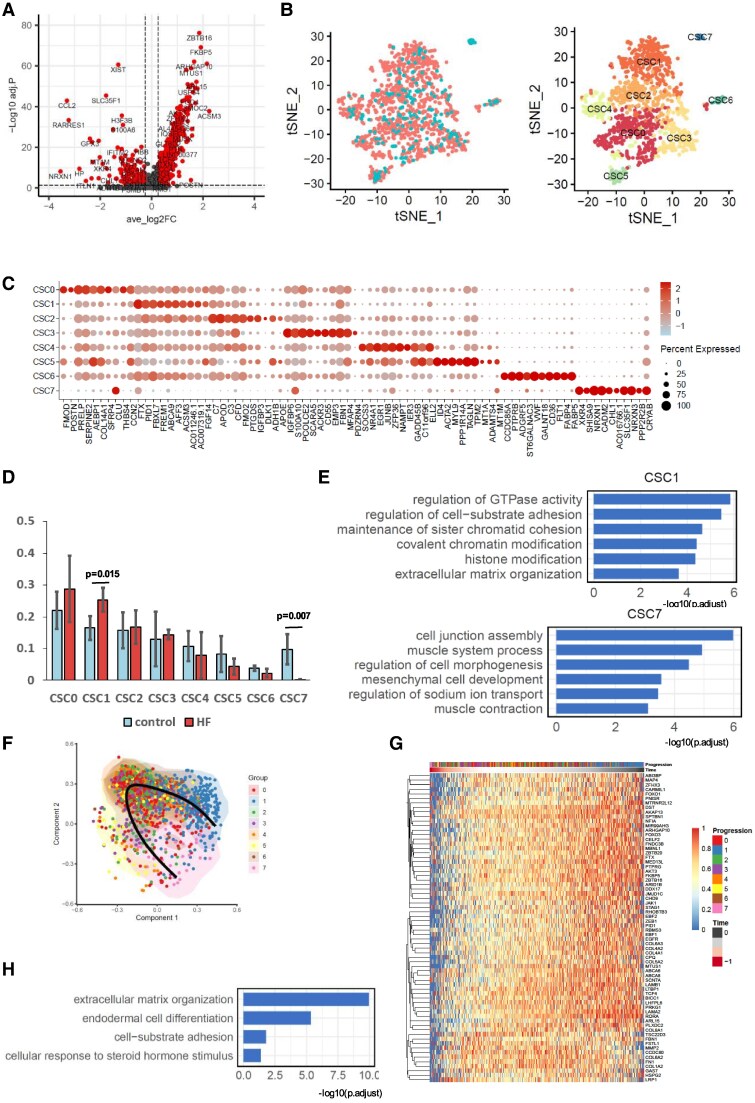
Cellular and subcellular *t*-distributed stochastic neighbour embedding clustering of cardiac stromal cells (CSCs) according to underlying disease. (*A*) Volcano plot for Differentially expressed gene between heart failure samples and control. (*B*) tSNE plot after integration and unsupervised clustering of both heart failure and control CSCs. (*C*) Dot plot of 10 marker genes for each CSC subcell cluster. (*D*) Sub cell type composition of CSC in both heart failure and control samples. (*E*) Selected gene ontologys for sub cell type clusters (CSC1 and CSC7). (*F*) Trajectory plot for fibroblast sub cell type transition from control to heart failure. (*G*) Heatmap for genes in Module 4 for trajectory analysis. (*H*) Selected gene ontologys for gene Module 4 gene set enrichment analysis

Previously, we have found that activation of FAP cells, the progenitor cells of tissue resident stromal cells, in skeletal muscle is associated with muscle fibrosis and regeneration.^26,27^ We speculated that the increasing CSCs population in the HFrEF hearts could share similar mechanism and pathogenesis with that in skeletal muscle. To address this, we next explored heterogeneity and differentiation trajectory of CSCs between the control and HFrEF hearts. We first compared the gene expression in the CSCs between control and HFrEF. We observed significant increase in the number of up-regulated genes than the number of down-regulated genes in HFrEF when compared with controls, suggesting an overall activation of gene expression in CSCs from HFrEF (*Figure 3A*). Gene ontology analysis based on the up-regulated genes further confirmed that the top six significantly enriched pathways (adjusted *P* value < 2e−09) are related to extracellular matrix (ECM) formation and transforming growth factor beta (TGFβ) signalling (Supplementary Table S3), suggesting the close link between CSCs and cardiac fibrosis.

Cardiac stromal cells are a population of highly heterogeneous stromal cell type.^27^ To gain further insight into the heterogeneity and differentiation trajectory of the CSCs over the progression of HFrEF, we performed unsupervised clustering of the CSCs based on single-cell transcriptome and identified eight distinct subpopulations (*Figure 3B*). Each CSC subtype expressed a group of distinct marker genes, as highlighted with dot plots in *Figure 3C*. Notably, two CSC Subtypes 1 and 7 were enriched in the HFrEF patients and non-HFrEF controls respectively (*Figure 3D*). To further elucidate the biological functions of these two CSC subtypes (Clusters 1 and 7), we performed GO enrichment analysis based on the enriched markers genes for each cluster. Our results indicated that CSCs in Cluster 1 were enriched in processes related to the regulation of GTPase activity, maintenance of sister chromatid cohesion, and ECM organization, which are important biological processes for biomass production and ECM formation, a feature of myofibroblasts.^28^ In contrast, the CSCs in Cluster 7 were enriched in biological processes associated with cell junction assembly, mesenchymal cell development, and muscle contraction (*Figure 3E*), indicating their potential progenitor role in maintaining cardiac muscle functions, resembling the phenotype of FAPs.

Based on the striking findings of the two distinct CSC subtypes, we speculated that during the progression of cardiac fibrosis, the FAPs (Cluster 7) was activated and differentiated into the ECM-forming myofibroblasts (Cluster 1). To investigate this, we performed lineage trajectory analysis based on the single-cell transcriptome of CSCs from control and HFrEF hearts. Indeed, pseudotime analysis revealed that CSC subtypes in Cluster 7 (FAPs) and Cluster 1 (myofibroblasts) represented the starting (early) and ending points (late) along the pseudotime trajectory, respectively (*Figure 3F*). We assessed gene importance along the trajectory and categorized genes into seven functional modules. One interesting observation was that the mitochondrial gene expression (Module 2) was first decreased, followed by gradual increase during the progression from FAPs into myofibroblasts (Supplementary Figure S3A). This suggests intermediate CSCs between FAPs and myofibroblasts are more glycolytic, which is a metabolic hallmark needed for biomass production associated with deposition of ECM and elevated oxidative stress. Concurrently, we noted an increase in ribosomal gene expression immediately after the early pseudotime, indicating an increase in protein synthesis and biomass production after FAPs activation (Supplementary Figure S3C). At the late pseudotime, both protein synthesis (Module 7) and production of contractile proteins (Module 6) were decreased (Supplementary Figure S3B–D). This reduction may contribute to the impeded mechanical function in HFrEF after fibrotic remodelling.

In addition to these three gene modules (Supplementary Figure S3), Module 4 contains genes exhibiting a continuously up-regulated expression during the differentiation from FAPs into myofibroblasts (*Figure 3G*). Gene ontology enrichment analysis of genes in Module 4 revealed that these genes are involved in ECM organization, endodermal cell differentiation and cellular response to steroid hormone stimulus (*Figure 3H*). These processes have known influences on CSCs behaviour and involve in ECM remodelling and fibrosis. Our results collectively suggest that activation of cardiac FAPs links to the progression of cardiac fibrosis in HFrEF.

### Isolation of CD34^±^CD45^−^CD31^−^ fibroadipogenic progenitors with fibrogenic and adipogenic differentiation potential from human hearts

**Figure 4:**
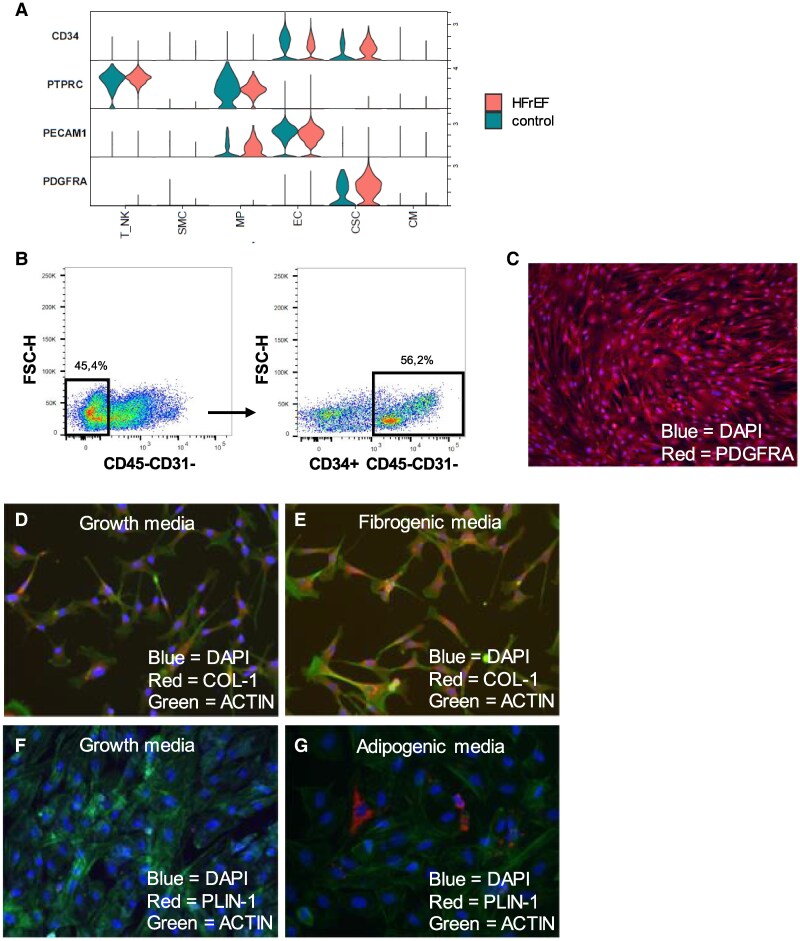
CD45^−^CD31^−^CD34^+^ cells isolated from the human myocardium possess fibro- and adipogenic differentiation potential. For subsequent experiments, mononuclear cells were isolated from the left ventricle of human hearts from heart transplantation recipients. CD45^−^CD31^−^CD34^+^ cells were further selected by fluorescence-activated cell sorting for *in vitro* experimentation. (*A*) *PDGFRA* is a marker of mesenchymal stem cells (multipotent stem cells) and was exclusively expressed in one cluster with a fibroblast phenotype. *CD34* was expressed in the same cluster as *PDGFRα*, as well as in a large cluster of *PECAM1* (CD31) positive cells, which represent endothelial cells. *PTPRC* (CD45) was exclusively expressed in two clusters representing macrophages and T/NK cells. (*B*) Live CD45^−^CD31^−^CD34^+^ cells isolated by fluorescence-activated cell sorting. (*C*) Isolated CD45^−^CD31^−^CD34^+^ cells can be grown in cell culture and express PDGFRα assessed by immunofluorescence staining (magnification 10X). (*D* and *E*) Isolated CD45^−^CD31^−^CD34^+^ cells increases COL-1 expression when grown in fibrogenic media, suggesting that they differentiate into fibroblasts. (*F* and *G*) Isolated CD45^−^CD31^−^CD34^+^ cells start to express PLIN-1 when grown in adipogenic media, suggesting that they differentiate into adipocytes. Thus, these cells may represent human cFAPs. The image magnifiation for D-G is 20X

We speculated that CSCs (activated FAPs) residing in the human myocardium represent a target for metformin, as the expression of the metformin transporter genes *SLC22A1*, *SLC22A3*, *SLC29A4*, and *SLC47A1* was elevated in CSCs from HFrEF compared with non-HFrEF controls (Supplementary Figure S4). To test this, we prospectively isolated progenitor cells (resting FAPs) from the left ventricle of explanted human HFrEF hearts from heart transplant recipients by FACS and used them for subsequent *in vitro* experimentation. Single-cell gene expression profiling reveals that *PDGFRA* is uniquely expressed in the CSC cluster (*Figure 4A*). However, PDGFRα is not reliably detectable on the surface of viable human stromal cells, preventing cell sorting of freshly isolated human cells using commercially available PDGFRα antibodies. We used negative selections for CD45 and CD31 to deplete immune and endothelial cells, respectively followed by positive selection for CD34 (*Figure 4A*), which is a commonly used to isolate human FAPs.^29,30^ Using FACS, we were able to sort live cardiac CD45^−^CD31^−^CD34^+^ FAP cells for *in vitro* experimentation (*Figure 4B*). We performed immunofluorescence staining of cultured cells to confirm that these cells indeed express PDGFRα (*Figure 4C*). When we exposed the cells to fibrogenic and adipogenic induction medium, they are highly capable of increasing COL-1 expression (*Figure 4D* and *E*) and increasing lipid droplet formation and perilipin-1 (PLIN-1) expression (*Figure 4F* and *G*). These data suggest that fibrogenic progenitors reside in the human myocardium possess stromal progenitor cell functions.

### The antifibrotic effect of metformin functions via inhibiting the proliferation of cardiac-derived activated fibroadipogenic progenitors

**Figure 5:**
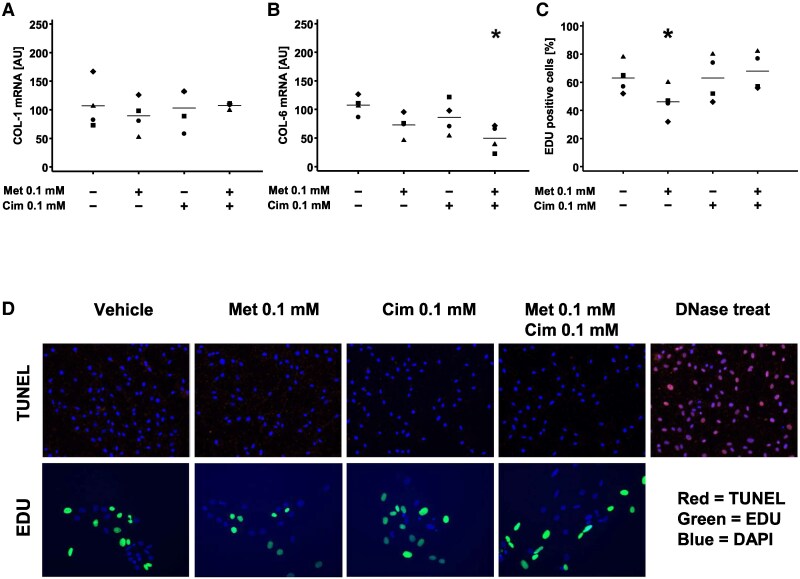
Metformin in pharmacological doses (0.1 mM) inhibits proliferation of fibrogenic progenitors. Mononuclear non-muscle cells were isolated from the left ventricle of explanted human hearts from heart transplantation recipients. Subsequently, CD45^−^CD31^−^CD34^+^ cells were isolated by fluorescence-activated cell sorting for *in vitro* experimentation. (*A* and *B*) *COL-1* and *COL-6* mRNA expression in cFAPs grown in fibrogenic media and incubated with metformin (0.1 mM) and cimetidine (0.1 mM) alone and in combination. (*C*) Quantification of proliferation assay as per cent 5-ethynyl-2'-deoxyuridine (EDU) positive cells. Individual donors (*A–C*) are indicated by the shape of the dots. (*D*) Cell proliferation assays (EDU) and apoptosis assays (TUNEL) performed on cFAPs in cell culture incubated with metformin (0.1 mM) and cimetidine (0.1 mM) alone and in combination (magnification 10X) (*P < .05)

Using the cardiac-derived FAPs, we next investigated the molecular and cellular mechanisms underlying the antifibrotic effect of metformin. We first evaluated if metformin could directly alter collagen production in mature fibroblasts. To address this, we first differentiated the FAP progenitors into mature fibroblasts and then treated them with metformin after differentiation. Quantitative gene expression analysis showed that metformin in pharmacological doses (0.1 mM) did not alter the mRNA level of *COL-1* or *COL-6*, the two major isoforms of collagen expressed in cardiac tissues (*Figure 5A* and *B*). COL-6 expression decreased during combined cimetidine and metformin treatment in combination, whereas the reductions observed with either agent alone did not reach statistical significance (*Figure 5B*). These findings indicate that metformin does not reduce mRNA level of *COL-1* and *COL-6* in FAP-derived fibroblasts, suggesting that the beneficial effect of metformin on counteracting cardiac fibrosis is independent of direct collagen production inhibition. We next evaluated if metformin affected the activation and proliferation of FAPs, which represents a potential mechanism by which fibrosis accumulates in the myocardium, by exposing freshly isolated FAPs to metformin in pharmacological doses (0.1 mM) and assessing cell proliferation. Our results showed that metformin significantly reduced FAP proliferation by ∼25%. This effect was completely abolished by co-incubation with cimetidine (0.1 mM) (*Figure 5C* and *D*), a competitive inhibitor of metformin transport through OCT1 and OCT3.^31^ Importantly, we confirmed that metformin and cimetidine in the applied doses did not induce apoptosis of the cells (*Figure 5C* and *D*), indicating that these compounds are not cytotoxic in the applied doses. Collectively, our results show that metformin in pharmacological doses has cardioprotective effects by counteracting cardiac fibrosis in HFrEF through inhibiting the activation and proliferation of cardiac-residing FAPs.

## Discussion

This study demonstrates that metformin inhibits activation and proliferation of cardiac progenitor cells with fibrogenic potential residing in human failing cardiac tissue from HFrEF patients. We thereby provide mechanistic evidence for pleotropic cardioprotective effects of metformin that may underlie improvements in heart failure-related outcomes in clinical trials.^32^ Our findings add to the growing body of evidence in favour of repurposing metformin for cardiovascular disease in patients without diabetes. However, before clinical application, the effect of metformin in HFrEF needs to be investigated in a large-scale randomized trial.^7^

Using different PET tracers, we demonstrate that metformin is only marginally distributed to the viable part of HFrEF myocardium, which aligns with previous observations in healthy humans.^24^ Thus, these results do not provide evidence for direct actions of metformin in mature cardiomyocytes. Instead, the inverse correlation between regional metformin net influx and myocardial viability—assessed by PET and echocardiography—suggests that metformin retention in HFrEF is confined to diseased tissue. The limited spatial resolution of PET prevents discrimination of subcellular tracer location, but compartment modelling of the obtained dynamic PET time activity curves may yield additional information about the intracellular fate of metformin. A compartment can represent a set of molecules that are bound to the tracer within it as a function of time. In HFrEF, a two-compartment model best describes metformin distribution in the heart. This suggests a reversible intracellular drug-target interaction without high-affinity intracellular binding of metformin. Metformin concentrations in diseased cardiac tissue are therefore unlikely to surpass concentrations in plasma, and this finding does not support accumulation of metformin in e.g. mitochondria.

Non-muscle cell–cell interactions have functional implications for cardiac development and repair.^33^ Thus, affecting non-muscle cells residing in the cardiac tissue may have implications for cardiac hypertrophy and remodelling in HFrEF. Due to limited access to human heart tissues, most of the current knowledge on cellular diversity rely on rodent heart tissue.^34,35^ Recently, cell types within different regions of the human heart have been revealed by single-cell seuqencing.^12^ Our scRNA-seq study contributes to a paradigm shift in our understanding of CSCs related to HFrEF. Within the non-myocyte cell pool, the CSCs (and FAPs) emerged as a diverse and influential cell type for cardiac remodelling and fibrosis. The observed heterogeneity within CSC subpopulations, influenced by underlying cardiomyopathy, suggests multifaceted roles for CSC in HFrEF pathophysiology. It remains to be addressed how the CSC subpopulations contributed to disease progression and underscores the need for tailored therapeutic strategies targeting specific fibroblast profiles.

Since pharmacological blockade of cardiac FAPs reduces post-myocardial infarction remodelling in mice,^36^ we hypothesized that non-muscle cell populations residing in the myocardium may represent targets of metformin. In our study, we demonstrate that cells with a fibroblast phenotype make up a large proportion of the mononuclear cell population in HFrEF. It should be appreciated that CSCs (fibroblasts) remain to be clearly defined as a cell type.^37^ In addition, we found that this cluster of CSCs represents a heterogeneous population of stromal cells with diverse phenotypes and functions. For example, *PDGFRα* is uniquely expressed in all cells that belong to this cluster, suggesting that it also contains mesenchymal progenitor cells. Indeed, our analysis identified a unique cluster of CSCs (Cluster 7) with progenitor functions as resting FAPs, which share many common features with FAPs in skeletal muscles.^26^ Of note, metformin has been observed to mitigate adipogenic differentiation of FAPs in skeletal muscle through activation of mTOR/ULK1-mediated autophagy.^19^

It is widely accepted that metformin inhibits fibrosis in several organs, including the lungs, kidneys, and ovaries.^18,38^ The exact mechanism responsible for these effects remains unclear. Our study provides evidence for a cellular target of metformin in HFrEF human hearts, thereby adding to our current understanding of cardioprotective effects observed in clinical ‘proof-of-concept’ studies. Metformin did not affect *COL1A1* and *COL6A1* mRNA levels in maturating fibroblasts derived from FAPs, at least for the culture cell models evaluated. Our results suggest that the mechanism of action for metformin’s antifibrotic effect appears to impede propagation of activated FAP (proliferation) rather than collagen production in already mature fibroblasts. This observation agrees with previous observations in mice, demonstrating that proliferation of FAPs is a major determinant of lipid and collagen accumulation in the diaphragm.^39^ Despite the extensive use of metformin over decades, no consensus exists on the expected plasma metformin levels during clinical use, but a systematic review of therapeutic plasma metformin concentrations in the literature identified concentrations ranging from 0.129 to 90 mg/l.^40^ Thus, metformin has functional effects on human fibrogenic progenitors in pharmacologically relevant doses. Moreover, the inhibitory effect of metformin on cellular proliferation was dependent on cellular uptake, as demonstrated by the ablated effect during co-incubation with inhibitors of uptake through OCT. The intracellular mechanism of action remains speculative. Previously, metformin has been shown to inhibit mammalian target of rapamycin complex 1 (mTORC1), a master regulator of cellular growth and proliferation, in neural progenitor cells in mice.^41^ Indeed, metformin has been suggested to inhibit cellular growth through multiple pathways,^42–45^ but common to all the proposed mechanisms is that they rely on experiments using supra-physiological doses of metformin. The data provided here incite future studies to investigate cellular mechanism of metformin using therapeutically relevant doses.

While this study was designed to explore direct effects of metformin on cardiac FAPs, the absence of ^11^C-metformin uptake in viable myocardium also suggest that indirect systemic mechanisms may underlie to the previously demonstrated improvements in myocardial efficiency and reverse remodelling.^5,6^ These may include enhanced endothelial and microvascular function^46^ and anti-inflammatory signalling,^47^ which can secondarily modulate cardiac metabolism and remodelling. Further studies are warranted to delineate these contributions.

In conclusion, metformin is marginally transported into less viable and fibrotic regions of the myocardium in HFrEF patients. Moreover, FAP cells reside in human HFrEF hearts, and clinically relevant doses of metformin inhibits proliferation of these cells *in vitro*. These findings provide mechanistic evidence for the pleiotropic cardioprotective effects of metformin observed in clinical trials.

### Limitations

First, the limited sample size in the PET study restricts statistical power and generalizability, although consistent absence of ^11^C-metformin uptake in viable myocardium supports robustness. Second, the tracer doses of metformin (∼10 µg) were far below therapeutic doses. Yet experimental data from a previous investigation does not suggest altered metformin distribution when administered at therapeutic doses.^48^ Third, the *in vitro* investigations of metformin-induced cellular effects are limited to non-ischaemic human cardiac tissue due to inaccessibility of ischaemic HFrEF tissue. However, cardiac expression levels of OCT have been shown to be similar between ischaemic and non-ischaemic HFrEF aetiologies in humans.^23^ Moreover, standard antifibrotic therapies for HFrEF demonstrate comparable effects and prognostic benefit in both DCM and ischaemic HFrEF.^49^

## Supplementary Material

xvag001_Supplementary_Data

## Declarations

### Data Availability

All data underlying the findings in this manuscript are provided as part of the article. Raw data on single cell gene expression have been deposited in the European Genome-phenome Archive (https://ega-archive.org) under access number GAS50000000125. The raw data that are not already presented in the figures are available from the corresponding author upon reasonable request.

### Pre-registered Clinical Trial Number

https://clinicaltrials.gov. Unique Identifier: NCT03122769.
