# Supplementary material for: Metformin inhibits proliferation of residing fibroadipogenic progenitor cells from failing human hearts
Source: ESC Heart Fail. 2026 Jan 22;13(1):xvag001. doi: 10.1093/eschf/xvag001 (PMC13108281; doi:10.1093/eschf/xvag001)
Supplement: xvag001_Supplementary_Data [file xvag001_supplementary_data.zip › Supplemental Material_251031.docx]

**Supplemental Material**

**Table of contents**

**Section A. Detailed Methods**

Study Design

Positron Emission Tomography

Echocardiography

Isolation of mononuclear cells from human heart tissue

Single-Cell Droplet-Based RNA Sequencing of mononuclear cells from human heart tissue

Public scRNA-seq data from left ventricle control hearts

ScRNA-seq data analysis

Fluorescence-activated cell sorting for *in vitro* experiments

Cellular characterization

Gene expression analysis by real time quantitative PCR

**Section B. Supplemental Figures**

(I) Figure S1. Quality control and clustering

(II) Figure S2. Reclustering and sub-celltype compositions in different samples

(III) Figure S3. Trajectory analysis for cardiac stromal cells

(IV) Figure S4. Heat map of relative metformin transporter genes expression across cardiac cells

**Section C. Supplemental Tables**

(I) Table S1. Characteristics for patients enrolled in the ^11^C-metformin biodistribution study

(II) Table S2. Key quality metrics of the barcoding and sequencing process

**Section D. Supplemental Data sets (Excel / CSV file format)**

(III) Table S3. Top 50 Cluster and sub-cluster marker genes

(IV) Table S4. Upregulated and downregulated genes in HFrEF

(V) Table S5. Gene list classified in modules for trajectory analysis

(VI) Gene ontology enrichment analysis

**Section A. Detailed Methods**

Study Design

This study contains data from two independent investigations of HFrEF patients. First, we investigated the myocardial biodistribution of ^11^C-metformin *in vivo* in HFrEF patients. Second, we used single cell RNA sequencing and *in vitro* response to metformin to characterize non-cardiomyocyte cells residing in the human heart.

Positron emission tomography

*^15^O-H_2_O PET*

Myocardial blood flow (ml min^-1^ g^-1^) was measured by PET to evaluate myocardial perfusion as previously described^1,2^. We used a Siemens Biograph TruePoint TrueV 64 PET/CT (Siemens Healthcare, Erlangen, Germany) after an initial low-dose CT (120 kV, 30 mAs, 4 mm slice thickness) for attenuation correction. A 6-min list-mode emission scan was started with simultaneous bolus injection of 400 MBq of ^15^O-H_2_O. PET emission data was reconstructed into a dynamic scan consisting of 22 time frames (1x10, 8x5, 4x10, 2x15, 3x20, 2x30, and 2x60 s) and a matrix size of 4x4x4 mm^3^ per frame. The TrueX reconstruction algorithm (three iterations, 21 subsets, post-filter 5 mm 3D Gaussian) was used, and dynamic data was analyzed using the aQuant Research software package^3^. We used a validated method for generation of parametric perfusable tissue index (PTI), defined as the ratio between perfusable and anatomical tissue fraction using a cut-off at 0.85 to evaluate myocardial viability^4,5^.

*^11^C-metformin PET*

Following the ^15^O-H_2_O PET, patients remained positioned in the PET/CT. Initial preparation of the ^11^C-metformin tracer was performed as previously described and contained 0.02-0.4 µg ml^-1^ metformin dissolved in aqueous diammonium phosphate (100 mM, pH 5)^6^. Each production was tested to >98% purity. Patients were injected with ^11^C-metformin and a 90-min list-mode emission scan covering an area from the ascending aorta to the epigastrium was obtained. Emission data was divided into 42 frames (12x5, 8x15, 7x60, and 15x300 s) and a matrix size of 4x4x4 mm^3^ per frame using the TrueX reconstruction algorithm (three iterations, 21 subsets, 3-mm Gaussian filter)^7^. Similar to the ^15^O-H_2_O scan, a low-dose CT scan was performed immediately before the PET. For delineation of the left ventricle, the ^15^O-H_2_O and ^11^C-metformin image volumes were co-registered using their respective low-dose CTs (rigid registration using the Regular Step Gradient Descent Optimizer implemented in Matlab Image Processing Toolbox (R2018b), the MathWorks Inc, Natick, Massachusetts) and the ^15^O-H_2_O delineation was transferred to ^11^C-metformin. Dynamic PET time activity curves were kinetically analyzed using plasma input functions in a two-tissue-compartment model with reversible binding. Goodness-of-fit was evaluated by the Akaike information criterium. The model derived K_1_ (mL mL^-1^ min^-1^), the influx rate constant of the tracer from the plasma to the tissue compartment; k_2_ (min^-1^), the rate constant of transfer of tracer out of the tissue; k_3_ (min^-1^), the rate constant of transfer into an intracellular compartment; and k_4_ (min^-1^), the transfer of tracer out of the intracellular compartment^7,8^. The metformin net influx rate (K_i_) (ml ml^-1^ min^-1^) was calculated as K_1_ x k_3_ x (k_2_ + k_3_)^-1^, which reflects the unidirectional uptake rate constant that incorporates both net inward transport and trapping of the radiotracer in the myocardium. All kinetic analysis was performed using the aQuant Research software package. Standardized uptake values (g mL^-1^) of ^11^C-metformin were calculated as the product of time activity concentration (kBq mL^-1^) and body weight (kg) divided by injected tracer dose (MBq).

Echocardiography

Echocardiographic examinations were performed prior to PET examinations in all patients at rest according to current guidelines^9^ and during exercise stress as previously described^10,11^. We used commercially available ultrasound systems (Vivid E9 and E95, GE Healthcare, Horten, Norway) equipped with a 3.5-MHz-phased array transducer (M5S) for two-dimensional (2D) evaluation. Images for evaluation of LV myocardial deformation were acquired as previously described^10^. Segmental myocardial contractile function was evaluated by two-dimensional speckle-tracking analysis (>60 frames/s) in a 17-segment model, producing estimates of global and segmental LV longitudinal strain (LS).

Isolation of mononuclear cells from human heart tissue

Immediately after removal, the explanted hearts were transported to the laboratory in ice-cold saline and 2-3 g of tissue was dissected from the left ventricle for isolation of mononuclear non-muscle cells. The hearts were transported from the operating room to the laboratory in less than 10 min. Under sterile conditions, the tissue was minced into small pieces with a scissor and transferred to HAMS-F10 media supplemented with 10% horse serum, 1% penicillin-streptomycin, 800 U ml^-1^ collagenase D (Bionordika, Cat. no. 214373) and 2.4 U ml_-1_ dispase II (Sigma, Cat. no. 4942078001) and gently minched for 1½ h at 37°C in a gentle-MACS Tissue Dissociator (Miltenyi Biotec). Digested samples were passed through a 70 µM filter to remove undigested material before cells were pelleted by centrifugation at 500 g. Cells were resuspended in StemMACS cryo-brew (Miltenyi Biotec, Cat. no. 130-109-558) and stored at -80°C and until Fluorescence-activated cell sorting (FACS) analysis.

Single-Cell Droplet-Based RNA Sequencing of mononuclear cells from human heart tissue

The frozen cells were thawed in 37^o^C water bath and immediately transferred to a tube containing 10 ml HAMS-F10 supplemented with 10% horse serum and 1% penicillin-streptomycin (wash buffer). After 5 min centrifugation at 500 g, the supernatant was removed, and cells were resuspended in wash buffer. Before FACS, Propidium iodide (PI) agent was added to stain non-viable cells (**Figure S1A**). PI negative single cell suspensions were converted to barcoded single cell RNA sequencing (scRNA-seq) libraries using Chromium Single Cell 3’ Library, Gel Dead & multiplex kit and Chip Kit (v3, 10x Genomics), aiming for 5,000 cells per library. Libraries were sequenced on an MGI-G400 sequencer aiming for 50,000 reads per cell.

Public scRNA-seq data from left ventricle control hearts

The scRNA-seq fastq files from left ventricle (LV) of control human heart (without HFrEF, hereafter referred as control) were collected from the Human Cell Atlas (HCA) Data Coordination Platform (DCP) with accession number: ERP123138. Four samples with high sequencing quality (including sequencing depth, MT percentage, features/counts per cell) were recruited, including CBTM-364B_LV_cells (ERR7423242), CBTM-390C_LV_cells (ERR7423265), CBTM-417C_LV_cells (ERR7423286) and CBTM-473C_LV_cells (ERR7423336).

ScRNA-seq data analysis

Raw data were mapped to the human genome (build GRCh38) using Cell Ranger software (10x Genomics, version 6-0.2). For all the samples, the following quality control steps were applied with Seurat R package (version4.0.3)^12^: (i) Genes expressed by less than three 3 cells were filtered; (ii) Cells that expressed either less than 200 (low quality cells) or more than 6000 genes (possible doublets), or more than 30% of UMIs derived from the mitochondrial genome were removed. Afterwards doublets were removed with DoubletFinder (version 2.0.3). Cell numbers before and after filtering are depicted in **Figure S1C**. After auto scaling, the normalized data from different patients were integrated and summarized by principal component analysis (PCA), the first 20 PCAs were used for further dimension reduction and visualized with t-Distributed Stochastic Neighbor Embedding (t-SNE). Major clusters of cells were identified by a shared nearest neighbor (SNN) modularity optimization-based clustering algorithm (resolution=0.2). To obtain ranked marker gene lists for each cluster, we performed pairwise differential gene expression analysis for each cluster against all other clusters independently, using the FindAllMarkers function in Seurat (parameter: only.pos = TRUE, min.pct = 0.25, logfc.threshold = 0.25).

*Sub cell-type classification*

For further classification of the major cell types into functionally distinct sub cell-types, cells from each major cell types were subsetted and clustered separately with different resolution. Data from different samples were split and re-integrated with CCA. PCA was performed on variable genes followed by t-SNE to construct a two-dimensional representation of the data. Sub-clusters were identified using the *FindClusters* function in Seurat. Positive markers for each sub-cluster were identified using the Seurat *FindAllMarkers* function, as described before. Differentially expressed gene analysis between two groups were analyzed with the FindMarkers function in Seurat.

*Gene ontology enrichment analysis*

The gene lists were subjected to gene ontology (GO) analysis using the enrichGO function implemented in the clusterProfiler package (v3.18.1). GO enrichment level was evaluated by adjusted P values, and multiple test adjustment was conducted using the Benjamini-Hochberg method.

*Pseudotime Analysis*

Trajectory analysis was performed with SCORPIUS (version 1.0.8). Briefly, normalized gene expression matrix were subjected to dimension reduction (ndim=5, num_landmarks=1000), followed by k-means clustering. Shortest path connecting all cluster centers were found with infer_trajectory function (k=4,maxit = 100,stretch=0). Genes importance for the trajectory were calculated with gene_importances function (num_permutations = 10, num_threads = 8, ntree = 10000, ntree_perm = 1000), and then classified into modules on the smoothed expression with extract_modules function.

*Data Visualization*

A range of visualization techniques were employed to effectively represent our findings. We generated tSNE plots, violin plots, and gene expression dot plots using the 'DimPlot', 'VlnPlot', and 'DotPlot' functions within the Seurat package. Additionally, heatmaps were created utilizing the ‘heatmap’ function in the stats package (version 4.0.3). Bar plot were created with ggplot2 (version 3.3.6). Volcano plot were created with EnhancedVolcano (version 1.8.0)

*Data availability*

Processed data are visualized in DREAMapp (<http://dreamapp.biomed.au.dk/heart_failure>).

Fluorescence-activated cell sorting for *in vitro* experiments

Frozen cells were thawed in 37^o^C water bath and immediately transferred to a tube containing 10 ml wash buffer. After 5 min centrifugation at 500 g, the supernatant was removed, and cells were resuspended in wash buffer. The cells were subsequently pelleted by centrifugation at 500 g. The supernatant was removed, and cells resuspended in 300 µl HAMS F10+ before 20 µl FcR blocking reagent (Miltenyi Biotec) and antibodies against CD45 (12 µl/sample), CD31 (4 µl/sample), and CD34 (20 µl/sample) were added (antibodies are specified in **Key Resources Table**). Samples were incubated with antibodies at 4°C in darkness for 30 min before 10 µl PI (Miltenyi Biotec) was added. Cell sorting was performed using a FACS-AriaIII cell sorter (BD Bioscience, San Jose, CA, USA) equipped with four lasers (405 nm, 488 nm, 561 nm, and 633 nm). Gating strategies were optimized through multiple earlier experiments, included various unstained, single color, and multiple color samples. Propidium Iodide (PI) was used remove dead cells. Live (PI^-^) CD45^-^CD31^-^CD34^+^ cells were collected and stored on ice until seeding in chamber slides. Not-stained cells and single-color controls were prepared in combination with the primary (full color) samples. To ensure bright single-color controls for compensation, compensation beads was utilized (specified in **Key Resources Table**). All subsequent *in vitro* experiments were performed without any passages to ensure the closest resemblance of the *in vivo* phenotype.

Cellular characterization

*Fibrogenic and adipogenic differentiation*

CD45^-^CD31^-^CD34^+^ cells were seeded in Poly-D-Lysine and extracellular matrix coated 12-well chamber slides and allowed to adhere for 48 h. Cells were then cultured in media (BioAMP2) and changed every 48 h reach >75% confluency. Subsequently, cells were grown two weeks in StemMACS AdipoDiff media to stimulate adipogenic differentiation or high-glucose Dulbecco’s modified eagle medium (DMEM) supplemented with 10% fetal bovine serum (FBS), and 1 ng/ml TGFβ to support fibrogenic differentiation. Evaluation of adipogenic differentiation was performed by immunostaining with Perilipin-1 (PLIN1) antibody, whereas evaluation of fibrogenic differentiation was performed by immunostaining with Collagen-1 (COL-1) antibody. Before immunostaining, cells were washed three times 5 min with PBS to remove the medium and subsequently fixed by incubation with 4% formaldehyde in PBS for 15 min at room temperature. Cells were blocked 20 min with 1% BSA in PBS supplemented with 10% FBS. Cells were then permeabilized by incubation with 0.5% Triton-X100 for another 20 min. Fixed cells were incubated overnight with primary antibodies at 4°C and incubated in secondary antibodies 1 h at room temperature (antibodies are specified in the **Key Resources Table**). F-Actin was labelled by incubation in ActinGreen Ready Probe Reagent (ThermoFisher) for 30 min at room temperature. Finally, cells were washed in PBS and incubated with DAPI for 5 min. COL-1 and PLIN1 were visualized using the EVOS M7000 imaging system (ThermoFisher).

*Cell proliferation assay*

Cells were seeded in Poly-D-Lysine and extracellular matrix coated 12-well chamber slides and allowed to adhere for 48 h. Subsequently, cells were grown another 72 h in growth medium and changed every 48 h. Cell proliferation was then determined using the Click-IT Plus EdU Imaging Assay (ThermoFisher, Cat. no. C10627) according to the protocol. In short, cells were incubated with Edu (20 µM) in growth medium supplemented with metformin (0.1 mM), cimetidine (0.1 mM) alone or in combination for 48 h. The cells were then fixed by incubation with 4% formaldehyde in PBS for 15 min at room temperature and permeabilized by incubation with 0.5% Triton X-100 in PBS for 20 min at room temperature. Fixed cells were incubated in Click-IT Plus reaction cocktail for 30 min protected from light. To visualize nucleus the cells were incubated with Hoechst for 30 min. The EVOS M7000 imaging system (ThermoFisher) was used to prepare images of the wells. Edu positive cells were counted using ImageJ software and presented as a percent of total cell population.

*Apoptosis assay*

Cells were seeded in Poly-D-Lysine and extracellular matrix coated 12-well chamber slides and allowed to adhere for 48 h. Subsequently, cells were grown another 72 h in growth medium and changed every 48 h. Apoptosis was then determined using the Click-IT TUNEL Alexa Flour Imaging Assay (ThermoFisher, Cat. no. C10247) according to the protocol. In short, cells were incubated in growth medium supplemented with metformin (0.1 mM), cimetidine (0.1 mM) alone or in combination for 48 h. The cells were then fixed by incubation with 4% paraformaldehyde for 15 min at room temperature and permeabilized by incubation with 0.5% Triton X-100 in PBS for 20 min at room temperature. A positive control was prepared by incubation of selected cells with DNase for 30 min to induce DNA strand breaks. Fixed cells were incubated in TdT reaction cocktail for 60 min at 37°C. Finally, cells were incubated in Click-IT reaction cocktail for 30 min protected from light. To visualize nucleus the cells were incubated with Hoechst for 30 min. The EVOS M7000 imaging system (ThermoFisher) was used to prepare images of the wells.

Gene expression analysis by real time quantitative PCR

The transcription of selected genes in CD45^-^CD31^-^CD34^+^ cells undergoing fibrogenic differentiation *in vitro* was analyzed by real time quantitative PCR. RNA was extracted using TRIzol (Gibco BRL/Life Technologies, Roskilde Denmark) using a mixer mill. The PCR-reactions were performed in duplicate using LightCycler SYBR Green master mix (Roche Applied Science) in a LightCycler 480 (Roche Applied Science) using the following protocol: One step at 95 °C for 3 min., then 95°C for 10 sec., 60°C for 20 sec. and 72°C for 10 sec and finally a melting curve analysis was performed. The increase in fluorescence was measured in real time during the extension step. The relative gene expression was estimated using the default “Advanced Relative Quantification” mode of the software version LCS 480 1.5.1.62 (Roche Applied Science) and specificity of the amplification was checked by melting temperature analysis. Primer pairs were designed using QuantPrime^13^ and are specified in **Key Resources Table**. The expression level of the housekeeping gene (β2 microglobulin) was similar between all groups and interventions. All primers were from DNA Technology (Risskov, Denmark). A similar set-up was used for negative controls, except that the reverse transcriptase was omitted and no PCR products were detected under these conditions

**References**

1. Harms HJ, Knaapen P, de Haan S, Halbmeijer R, Lammertsma AA, Lubberink M. Automatic generation of absolute myocardial blood flow images using [15O]H2O and a clinical PET/CT scanner. *European journal of nuclear medicine and molecular imaging*. 2011;38:930-939. doi: 10.1007/s00259-011-1730-3

2. Clemmensen TS, Eiskjaer H, Logstrup BB, Mellemkjaer S, Andersen MJ, Tolbod LP, Harms HJ, Poulsen SH. Clinical features, exercise hemodynamics, and determinants of left ventricular elevated filling pressure in heart-transplanted patients. *Transpl Int*. 2016;29:196-206. doi: 10.1111/tri.12690

3. Harms HJ, Hansson NHS, Kero T, Baron T, Tolbod LP, Kim WY, Frokiaer J, Flachskampf FA, Wiggers H, Sorensen J. Automatic calculation of myocardial external efficiency using a single (11)C-acetate PET scan. *J Nucl Cardiol*. 2018;25:1937-1944. doi: 10.1007/s12350-018-1338-0

4. Knaapen P, Boellaard R, Gotte MJ, van der Weerdt AP, Visser CA, Lammertsma AA, Visser FC. The perfusable tissue index: a marker of myocardial viability. *Journal of nuclear cardiology : official publication of the American Society of Nuclear Cardiology*. 2003;10:684-691.

5. Timmer SAJ, Teunissen PFA, Danad I, Robbers L, Raijmakers P, Nijveldt R, van Rossum AC, Lammertsma AA, van Royen N, Knaapen P. In vivo assessment of myocardial viability after acute myocardial infarction: A head-to-head comparison of the perfusable tissue index by PET and delayed contrast-enhanced CMR. *Journal of nuclear cardiology : official publication of the American Society of Nuclear Cardiology*. 2017;24:657-667. doi: 10.1007/s12350-015-0329-7

6. Jakobsen S, Busk M, Jensen JB, Munk OL, Zois NE, Alstrup AK, Jessen N, Frokiaer J. A PET Tracer for Renal Organic Cation Transporters, (1)(1)C-Metformin: Radiosynthesis and Preclinical Proof-of-Concept Studies. *Journal of nuclear medicine : official publication, Society of Nuclear Medicine*. 2016;57:615-621. doi: 10.2967/jnumed.115.169292

7. Gormsen LC, Sundelin EI, Jensen JB, Vendelbo MH, Jakobsen S, Munk OL, Hougaard Christensen MM, Brosen K, Frokiaer J, Jessen N. In Vivo Imaging of Human 11C-Metformin in Peripheral Organs: Dosimetry, Biodistribution, and Kinetic Analyses. *Journal of nuclear medicine : official publication, Society of Nuclear Medicine*. 2016;57:1920-1926. doi: 10.2967/jnumed.116.177774

8. Sundelin E, Gormsen LC, Jensen JB, Vendelbo MH, Jakobsen S, Munk OL, Christensen M, Brosen K, Frokiaer J, Jessen N. Genetic Polymorphisms in Organic Cation Transporter 1 Attenuates Hepatic Metformin Exposure in Humans. *Clinical pharmacology and therapeutics*. 2017;102:841-848. doi: 10.1002/cpt.701

9. Lang RM, Badano LP, Mor-Avi V, Afilalo J, Armstrong A, Ernande L, Flachskampf FA, Foster E, Goldstein SA, Kuznetsova T, et al. Recommendations for cardiac chamber quantification by echocardiography in adults: an update from the American Society of Echocardiography and the European Association of Cardiovascular Imaging. *Eur Heart J Cardiovasc Imaging*. 2015;16:233-270. doi: 10.1093/ehjci/jev014

10. Larsen AH, Clemmensen TS, Wiggers H, Poulsen SH. Left Ventricular Myocardial Contractile Reserve during Exercise Stress in Healthy Adults: A Two-Dimensional Speckle-Tracking Echocardiographic Study. *Journal of the American Society of Echocardiography : official publication of the American Society of Echocardiography*. 2018. doi: 10.1016/j.echo.2018.06.010

11. Larsen AH, Jessen N, Nørrelund H, Tolbod LP, Harms HJ, Feddersen S, Nielsen F, Brøsen K, Hansson NHS, Frøkiær J, et al. A randomized, double-blind, placebo-controlled trial of metformin on myocardial efficiency in insulin resistant chronic heart failure patients without diabetes. *Eur J Heart Fail*. 2019;(in press) 10.1002/EJHF.1656. doi: 10.1002/EJHF.1656

12. Satija R, Farrell JA, Gennert D, Schier AF, Regev A. Spatial reconstruction of single-cell gene expression data. *Nat Biotechnol*. 2015;33:495-502. doi: 10.1038/nbt.3192

13. Arvidsson S, Kwasniewski M, Riano-Pachon DM, Mueller-Roeber B. QuantPrime--a flexible tool for reliable high-throughput primer design for quantitative PCR. *BMC Bioinformatics*. 2008;9:465. doi: 10.1186/1471-2105-9-465

**Section B. Supplemental Figures**

**Figure S1. Quality control and clustering**

(A) Live single cells (PI-) were identified by FACS and selected for RNA barcoding using the 10X Genomics platform. Cells isolated from four donors were analysed (HFrEF Patient 1-4)

(B) Violin plots showing the number of genes (nFeature) and UMIs (nCount), and percentage mitochondrial reads (percent.mt) for all cells in the filtered data set.

(C) The number of cells before filtering (red) and after firstly filtering of dead cells and dying cells (high percentage of mitochondrial gene expression, green) and then filtering of doublets by DoubletFinder (blue) for each of the samples

(D) t-SNE plots of single cell RNA sequencing data from all samples

(E) Cell type composition in different samples as percentage and absolute numbers


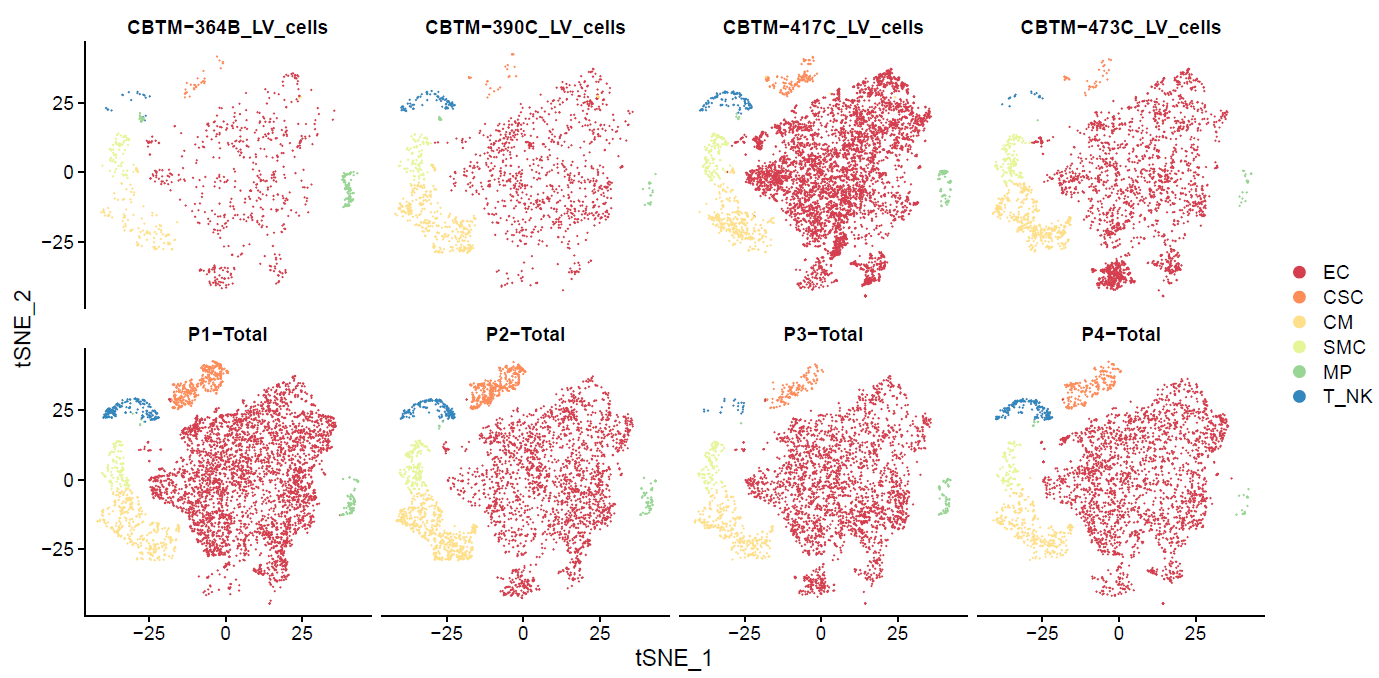

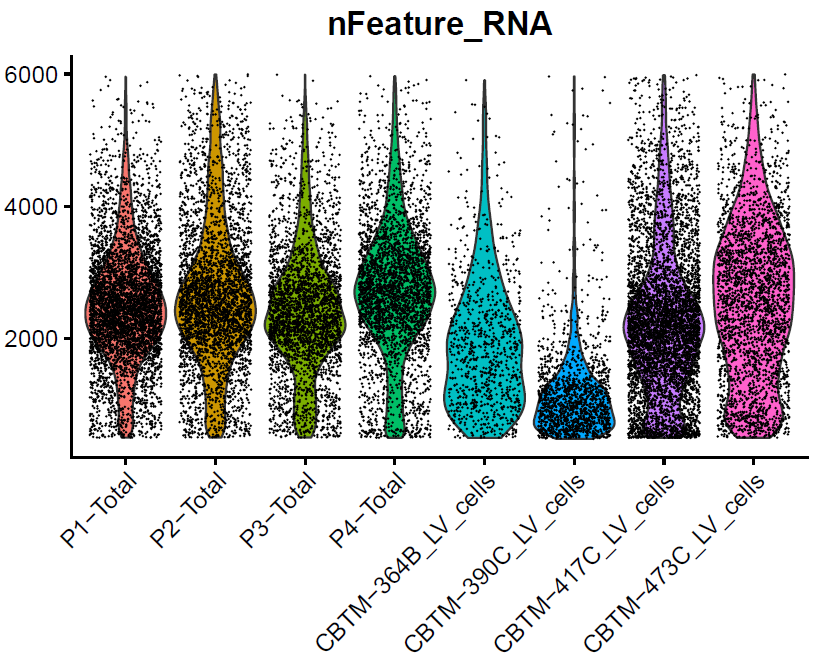

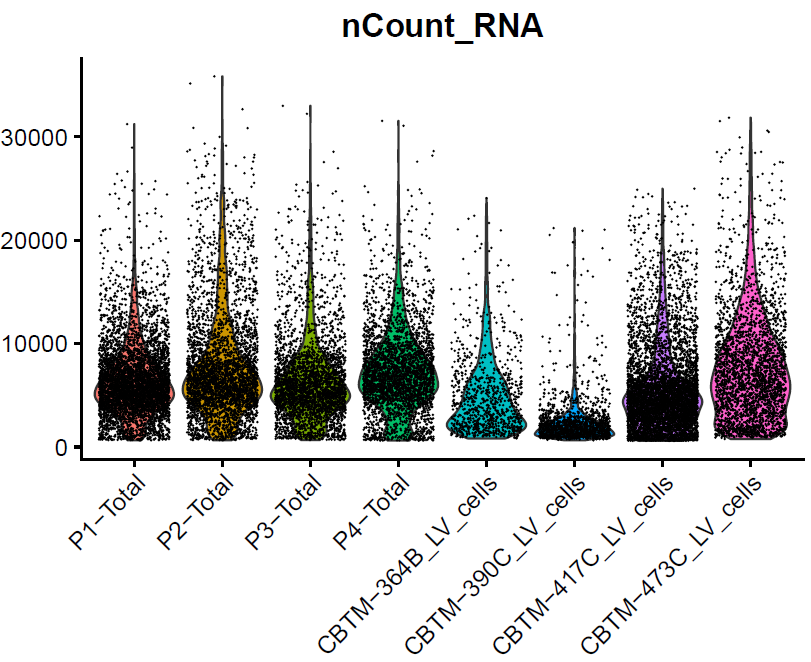

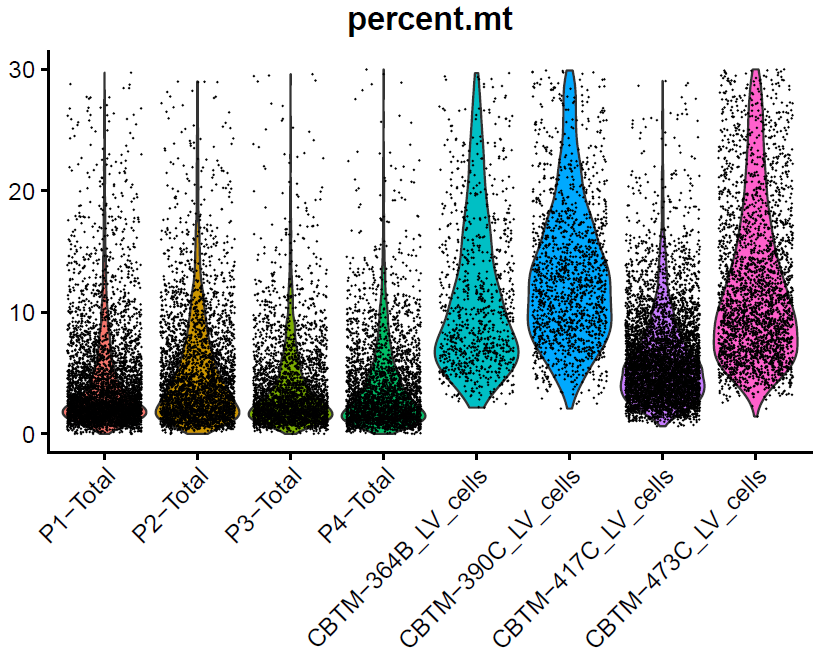


**A**

**B**

**C**


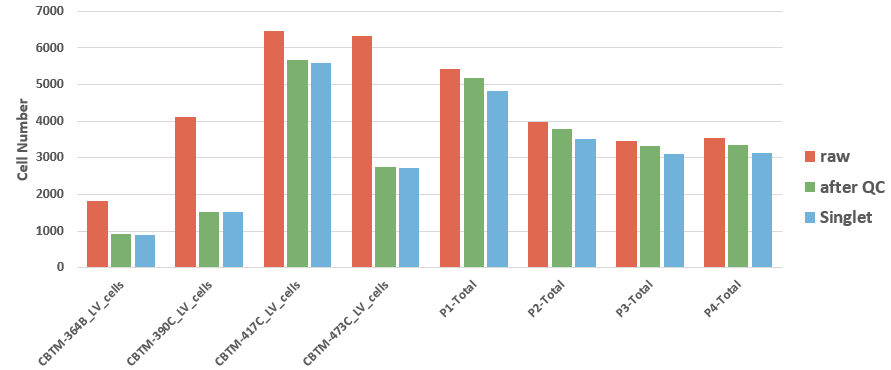


**D**

**E**


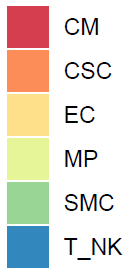

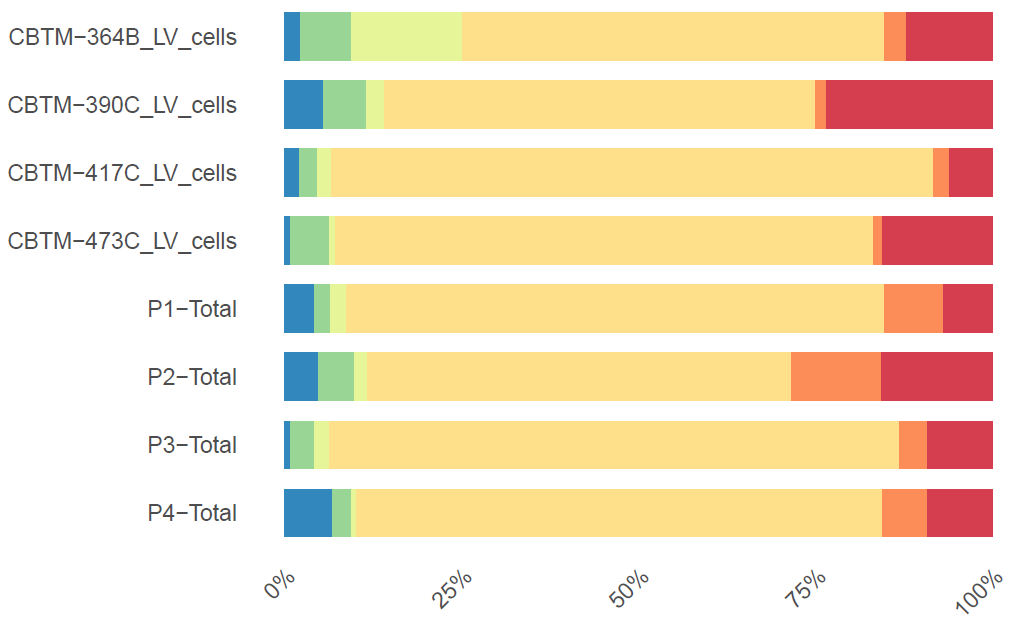

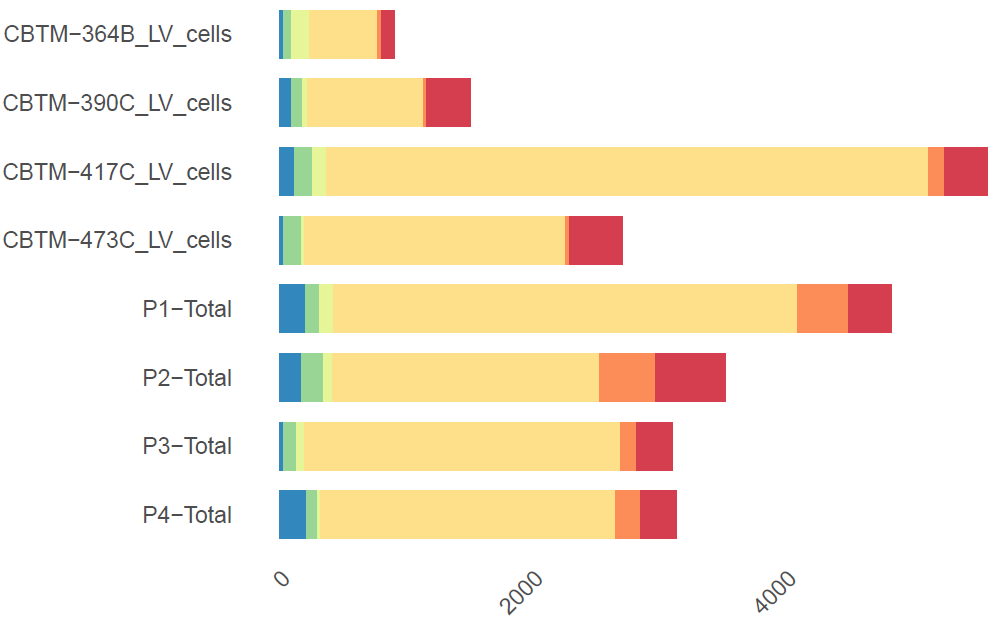

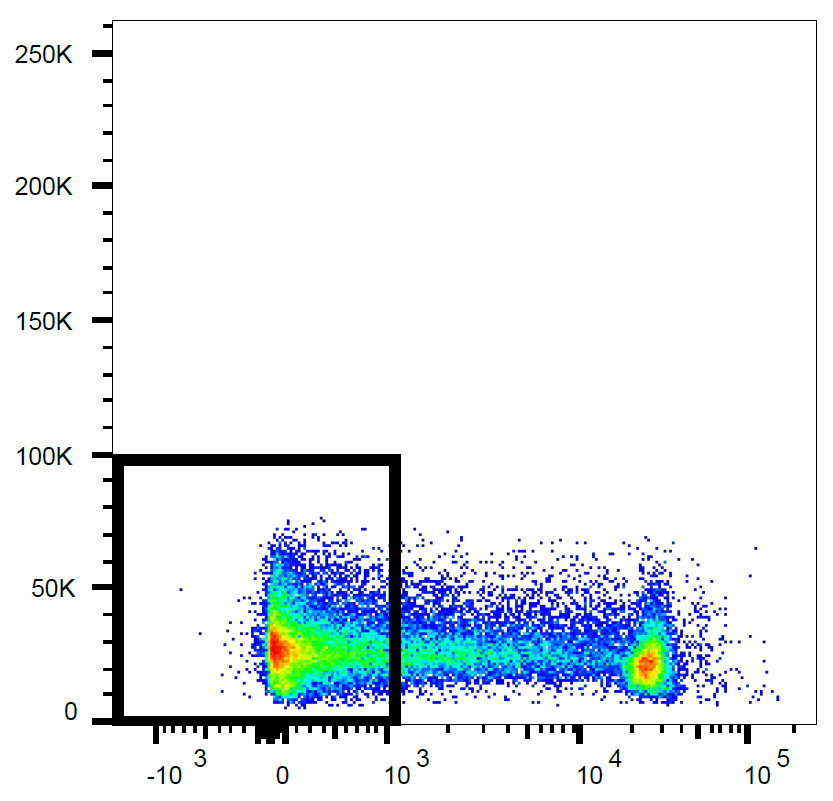


Alive cells

56,4%


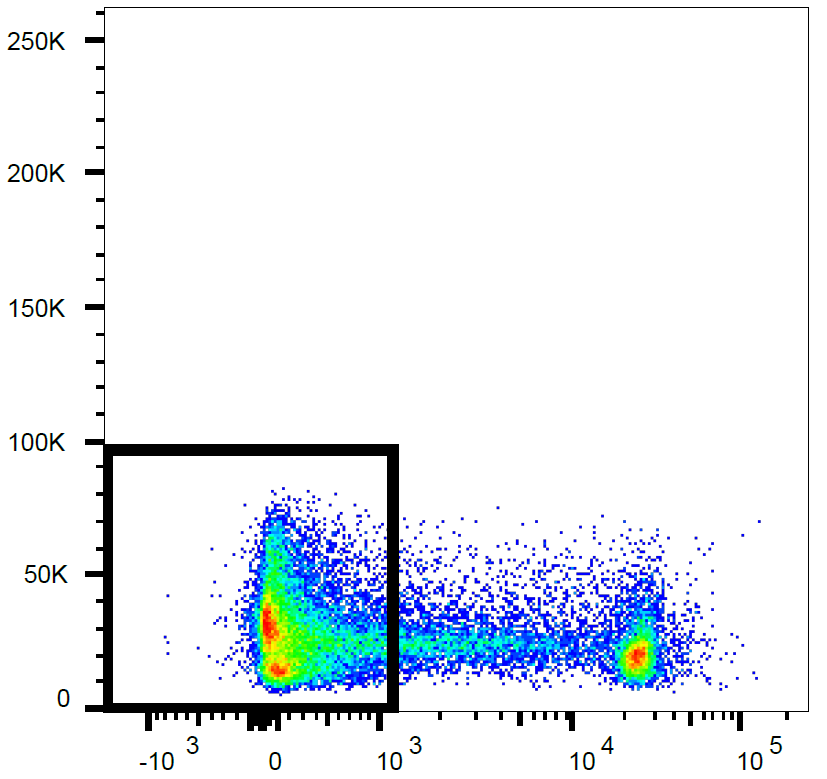

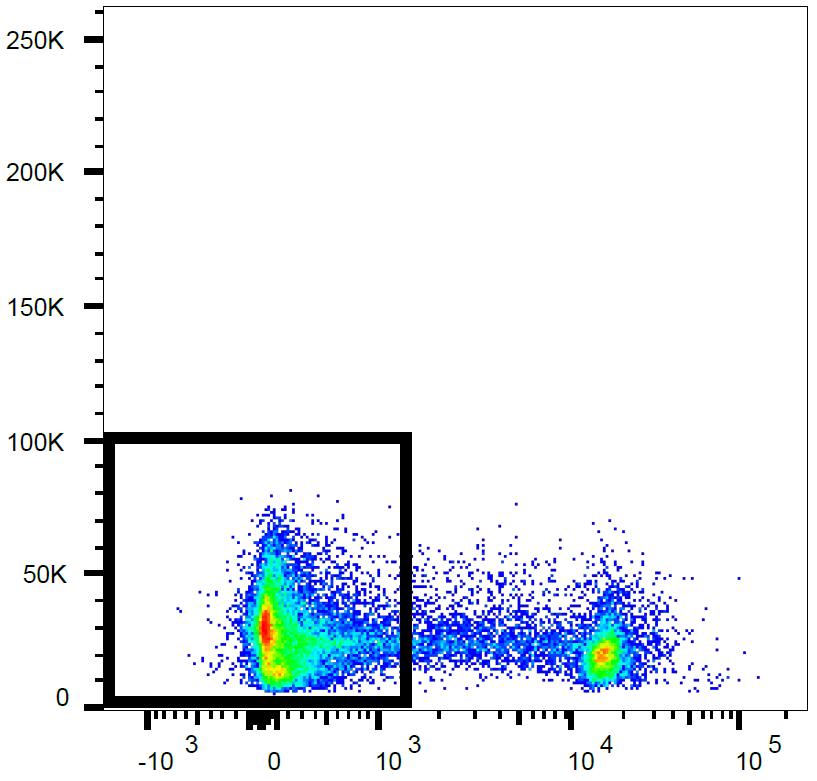

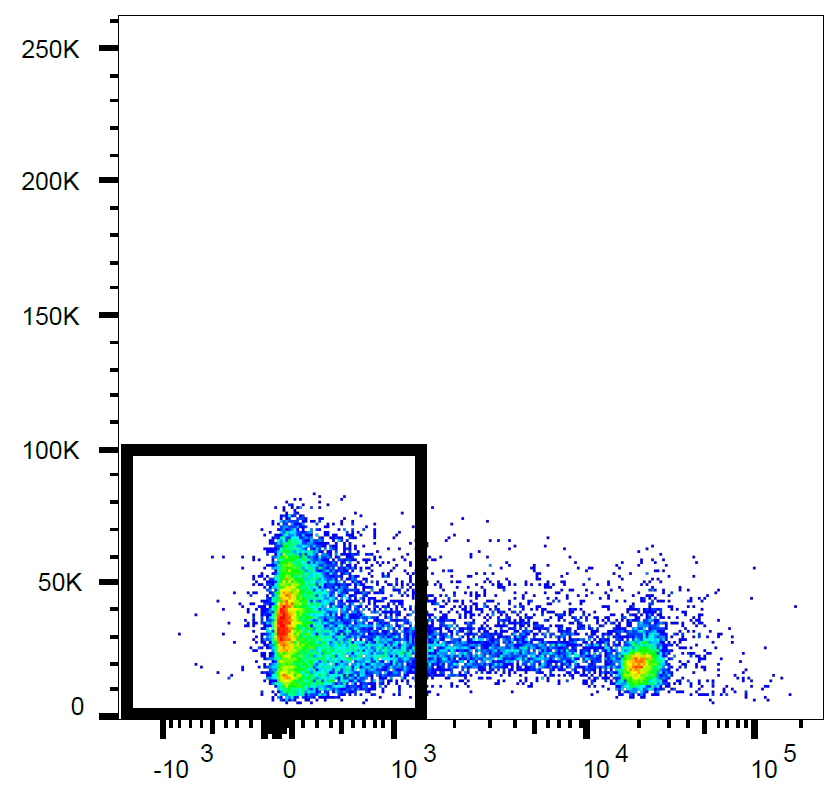


**Patient 1**

**Patient 2**

**Patient 3**

**Patient 4**

Alive cells

66,1%

Alive cells

70,7%

Alive cells

71,8%

**FSC-H**

**PI**

**Figure S2. Reclustering and sub-celltype compositions in different samples**

CM


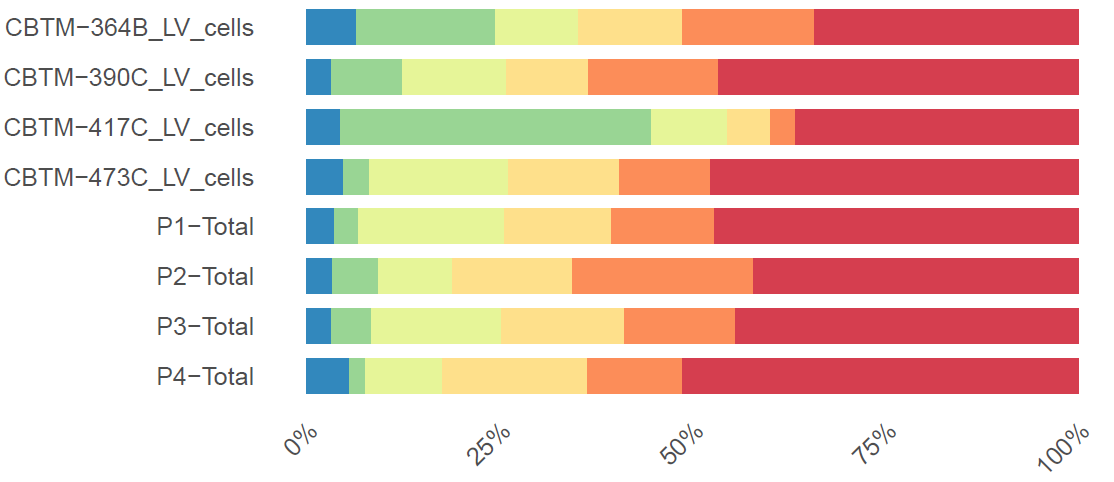

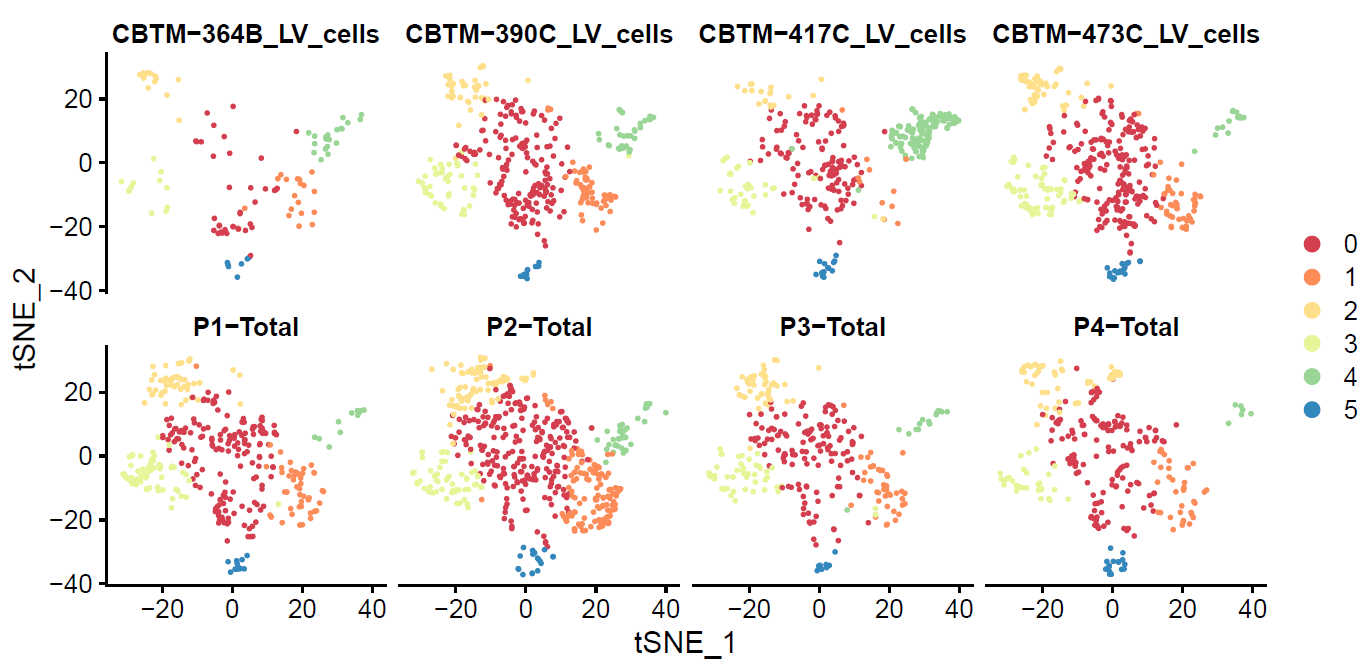

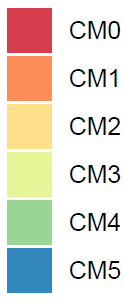

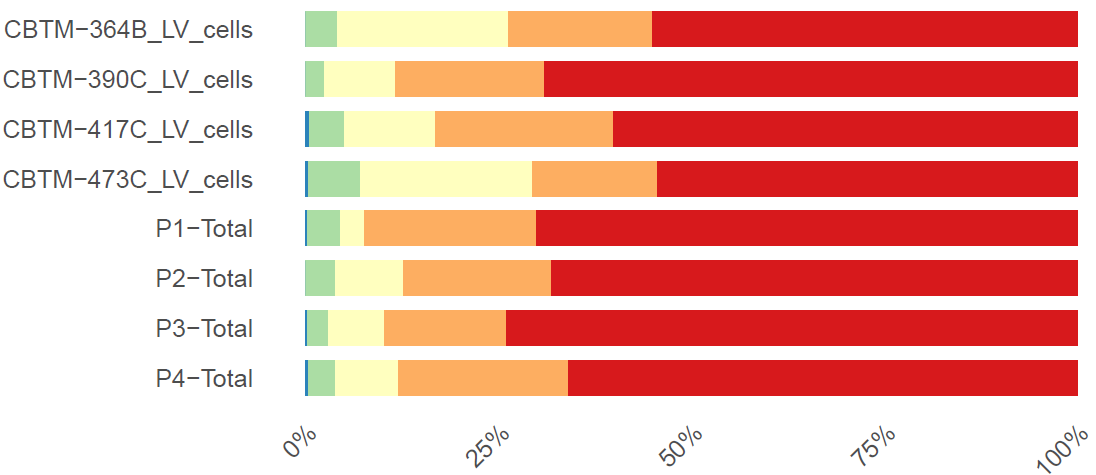

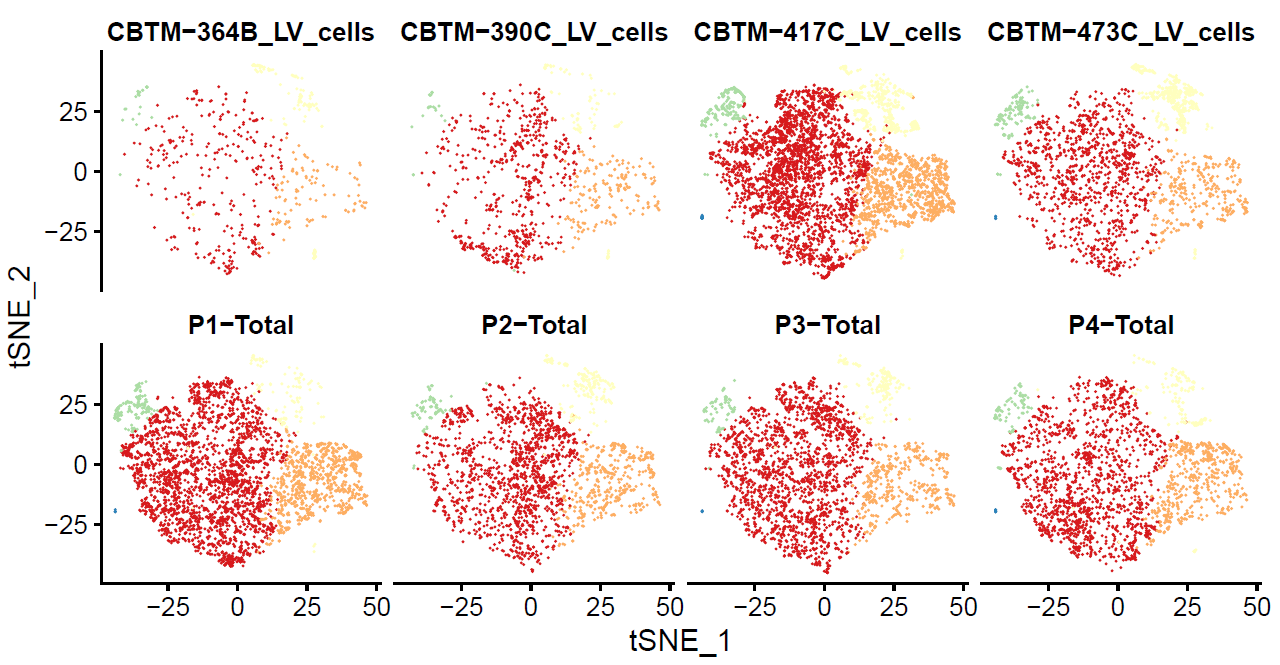

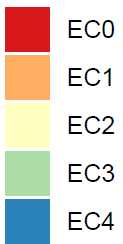


EC


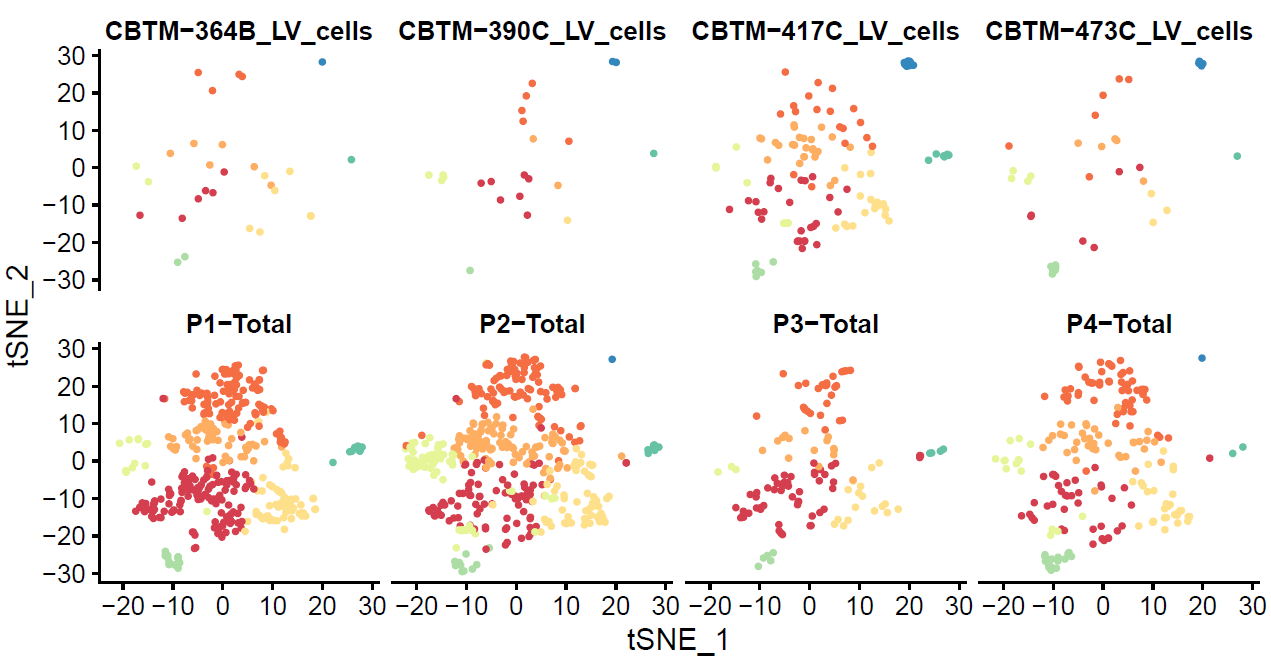

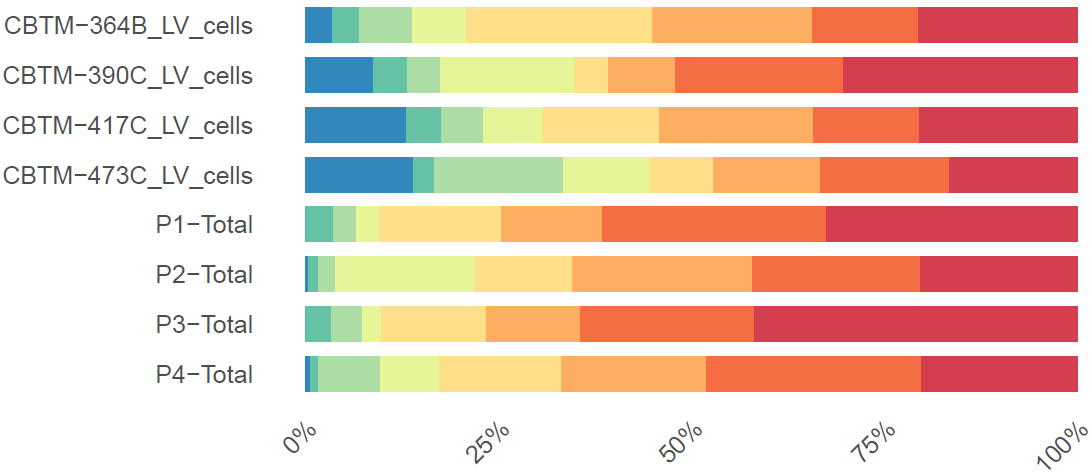


CSC


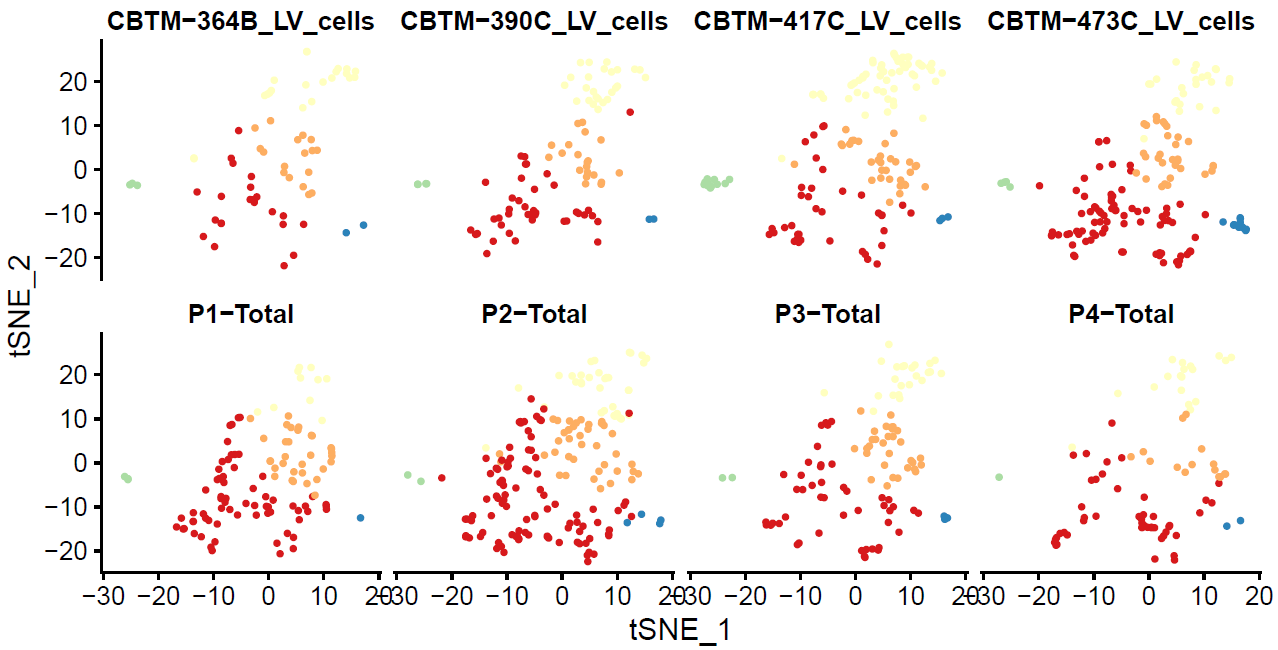


SMC


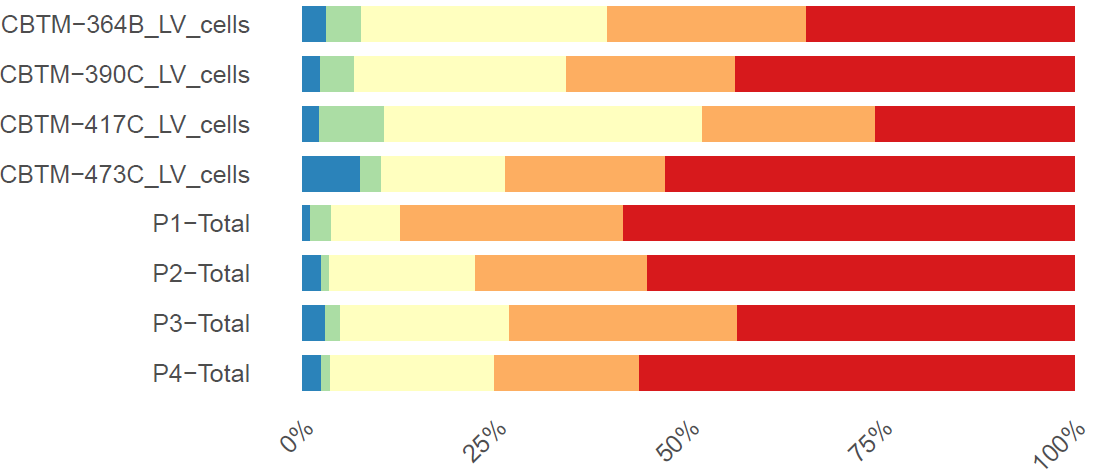

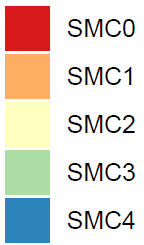

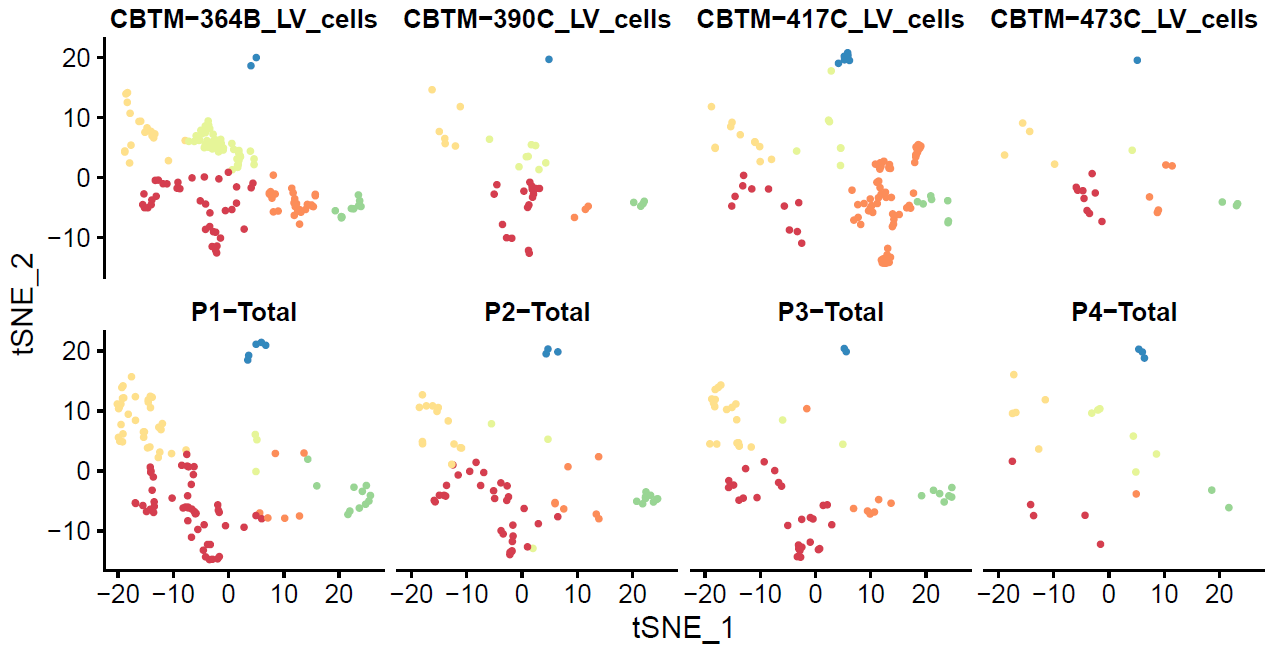


MP


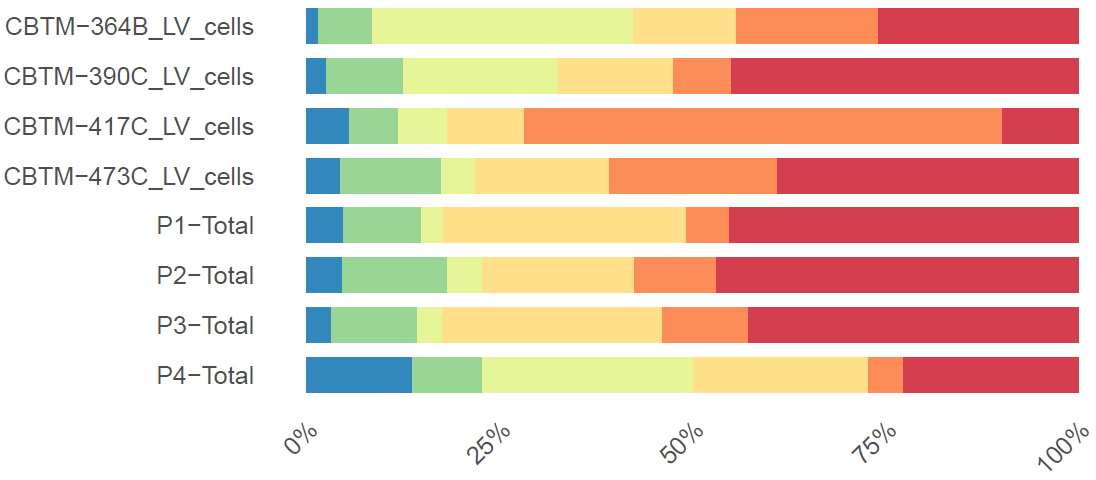

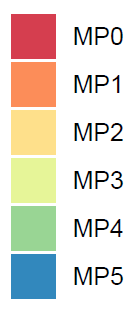

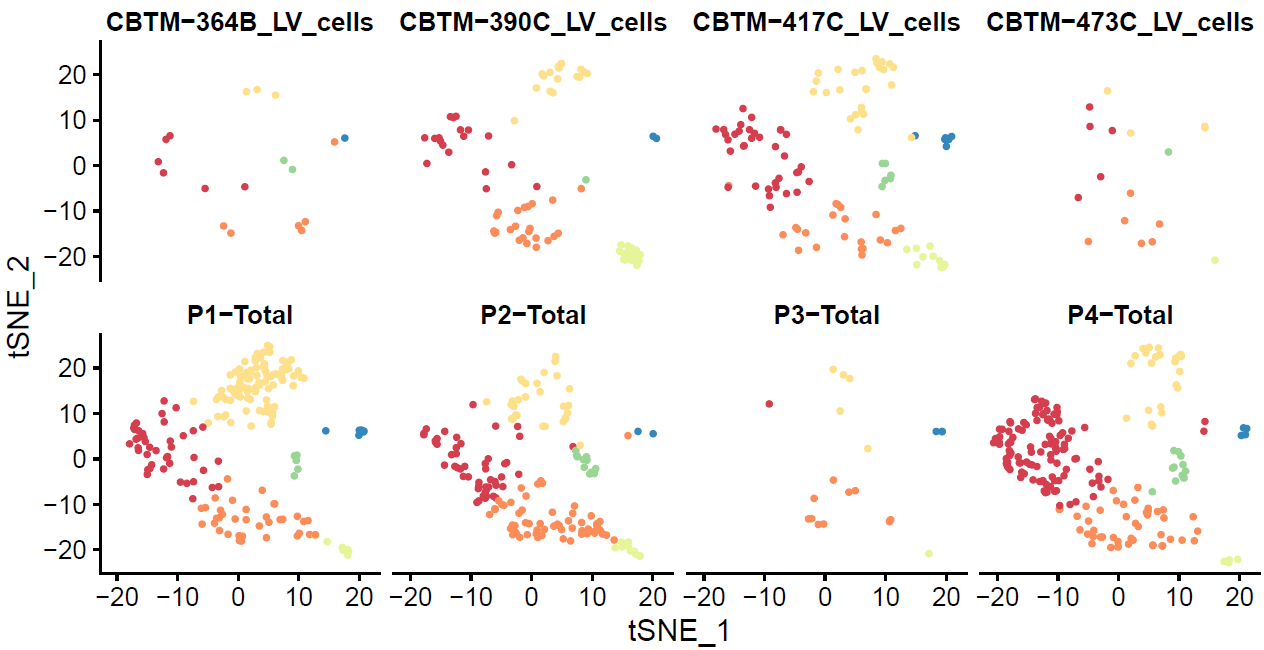

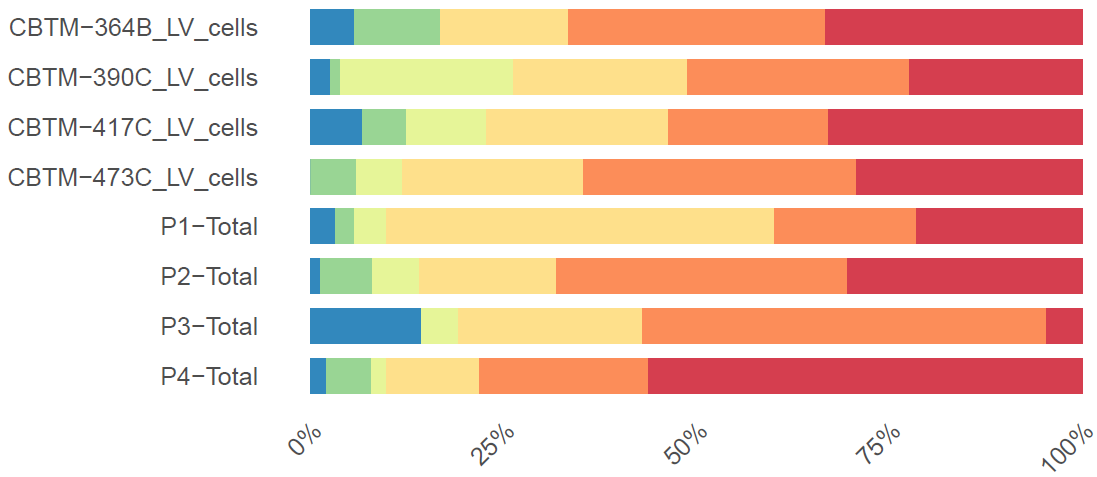

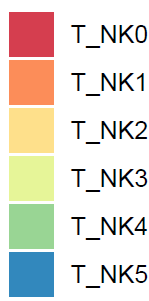


T_NK


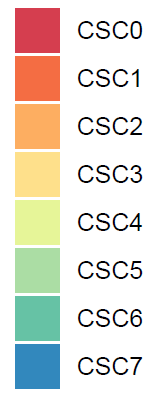


**Figure S3. Trajectory analysis for cardiac stromal cells**


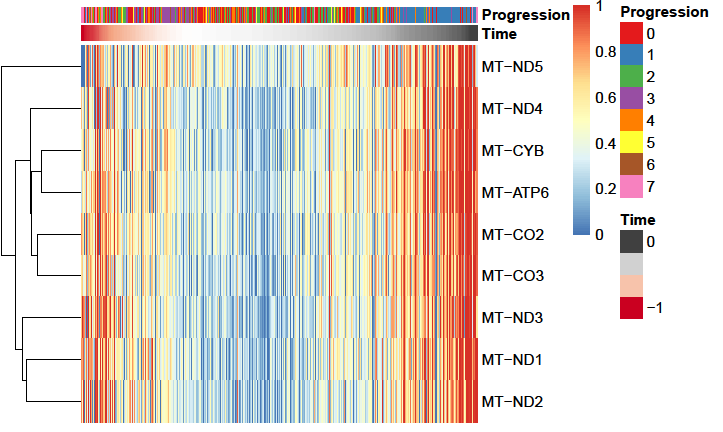

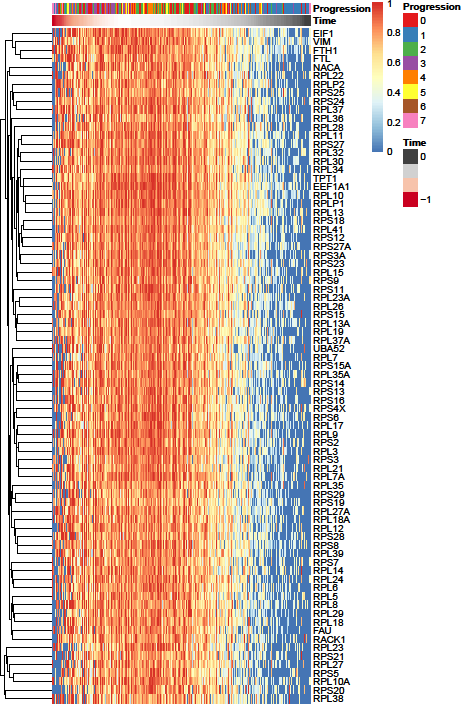

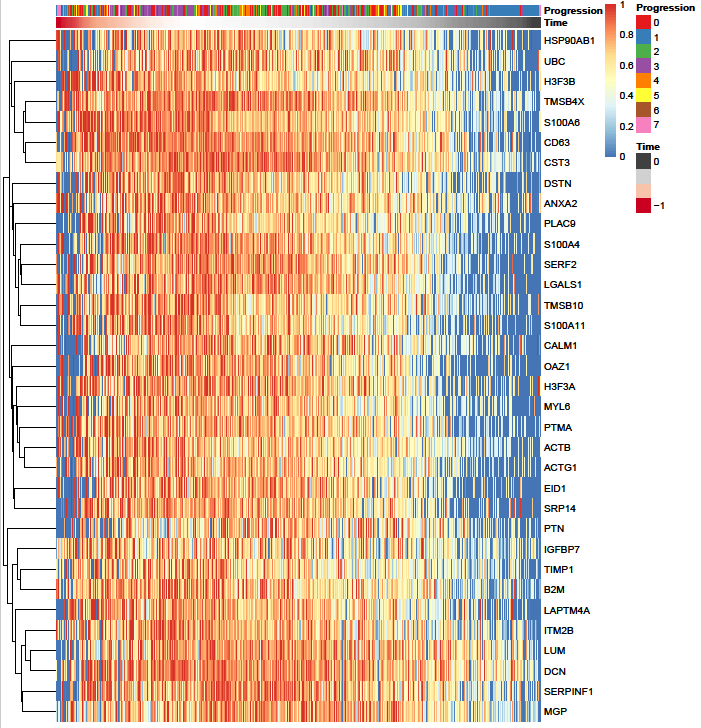


Module 2

Module 7

Module 6

**A**

**B**

**C**


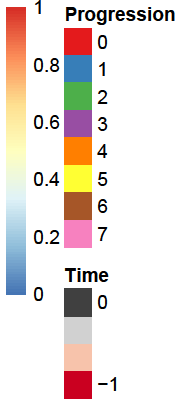


**D**


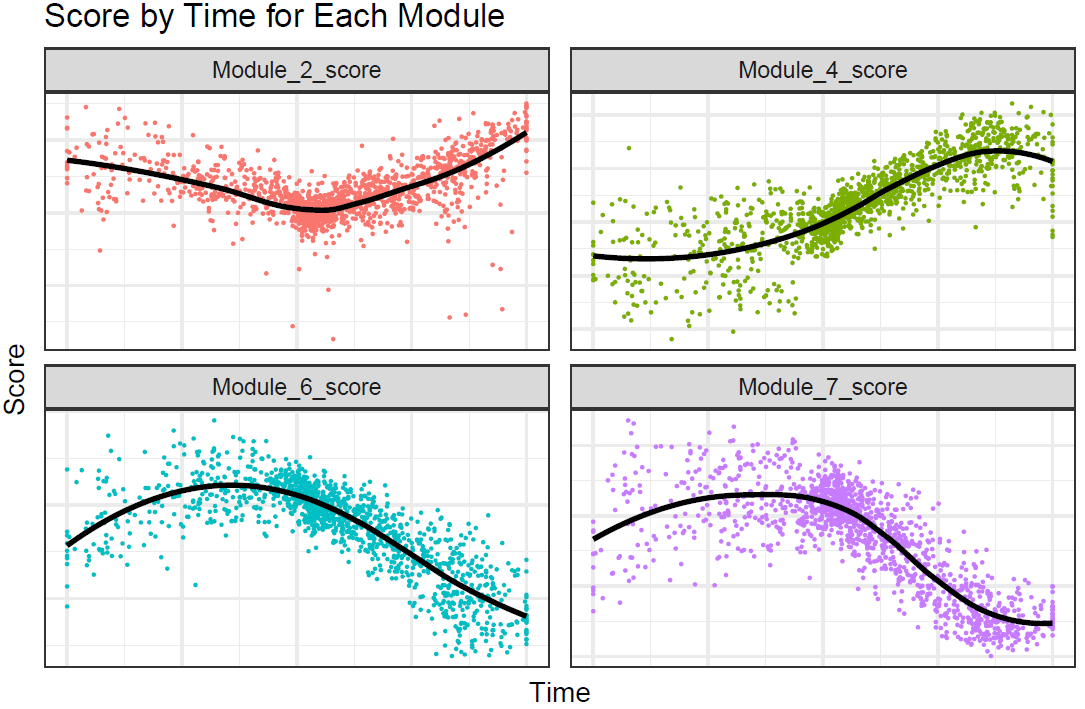


(A-C) Gene expression patterns during the progression from FAPs (cluster 7) into myofibroblasts (cluster 1) for genes in module 2 (mitochondrial genes), module 6, and module 7 (mostly ribosomal genes).

(D) Gene set score for gene module 2, 4, 6 and 7 for each of the cells in pseudotime.

**Figure S4. Heat map of relative metformin transporter genes expression across cardiac cell types**

**
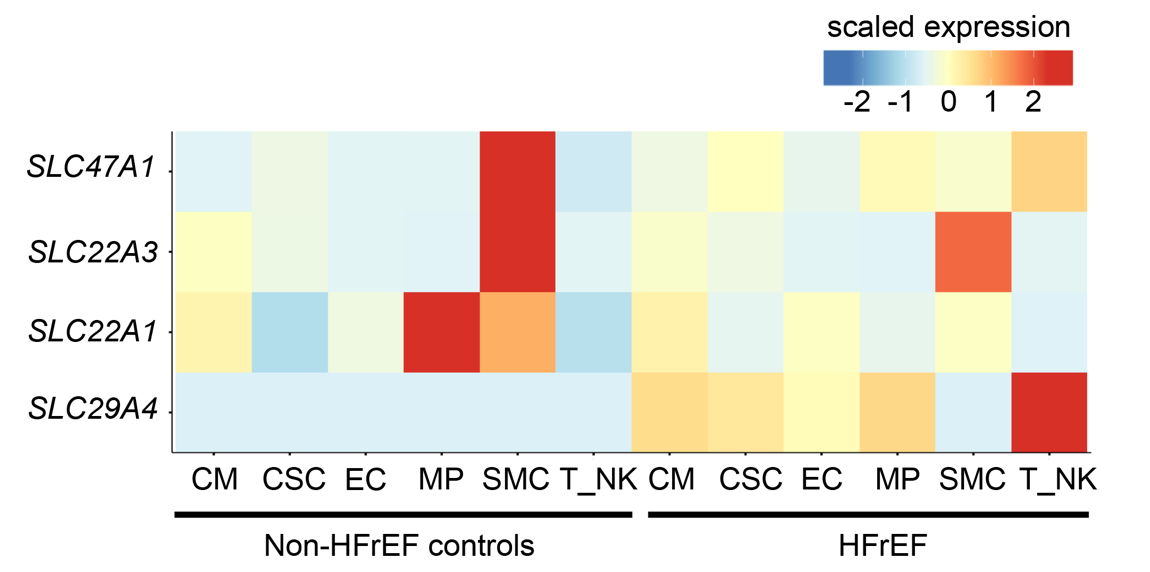
**

Relative gene expression for SLC22A1, SLC22A3, SLC29A4, and SLC47A1 in cells from non-heart failure controls (non-HFrEF) and failing human heart (HFrEF) tissues, including cardiomyocytes (CM), cardiac stromal cells (CSC), endothelial cells (EC), macrophages (MP), smooth muscle cells (SMC), and T/NK cells (T_NK).

**Section C. Supplemental Tables**

**Table S1.** Characteristics for patients enrolled in the ^11^C-metformin biodistribution study

|  | Patients (n = 7) |
| --- | --- |
| **Characteristics** |  |
| Age, yrs | 67 (66 to 74) |
| Males | 7 (100%) |
| BMI, kg m^-2^ | 26 ± 3 |
| Systolic blood pressure, mmHg | 121 ± 20 |
| Diastolic blood pressure, mmHg | 75 ± 18 |
| Heart rate, bpm | 60 ± 8 |
| NYHA II/III | 5 (71%) / 2 (29%) |
| **History** |  |
| Myocardial infarction | 7 (100%) |
| CABG | 3 (43%) |
| PCI | 4 (57%) |
| Atrial fibrillation | 4 (57%) |
| Hypertension | 4 (57%) |
| COPD | 1 (14%) |
| Smoking | 6 (86%) |
| **Left ventricular function** |  |
| LV EF, % | 36 ± 8 |
| LV GLS, % | 11 ± 2 |
| **Treatment** |  |
| ACE inhibitor / ARB | 7 (100%) |
| Beta-blocker | 6 (86%) |
| MRA | 4 (57%) |
| Diuretic | 4 (57%) |
| Statin | 6 (86%) |
| CRT | 4 (57%) |
| **Biochemistry** |  |
| HbA1c, mmol mol^-1^ | 40.3 ± 2.7 |
| Fasting glucose, mmol L^-1^ | 5.9 ± 0.5 |
| NT-proBNP, nmol L^-1^ | 562 (164 to 847) |

Data are presented as mean ± standard deviation, n(%), or median (interquartile range). BMI, body mass index; bpm, beats per minute; CABG, coronary artery bypass grafting; PCI, percutaneous coronary intervention; COPD, Chronic obstructive pulmonary disease; NYHA, New York Heart Association; LV, left ventricular, EF, ejection fraction; GLS, global longitudinal strain; CI, cardiac index; ACE, angiotensin-converting enzyme; ARB, angiotensin receptor blocker; MRA, mineralocorticoid receptor antagonist; CRT, cardiac resynchronization therapy.

**Table S2.** Key quality metrics of the barcoding and sequencing process

| **Group** | **Sample ID** | **Number of reads** | **Saturation^*^** | **Number of cells** | **UMIs/cell^†^** | **Genes/cell^‡^** |
| --- | --- | --- | --- | --- | --- | --- |
| HFrEF | P1-Total | 172,593,458 | 62.7% | 5,428 | 5,878 | 2,463 |
|  | P2-Total | 164,563,784 | 65.8% | 3,972 | 6,589 | 2,559 |
|  | P3-Total | 162,254,342 | 75.6% | 3,459 | 5,648 | 2,370 |
|  | P4-Total | 157,550,946 | 71.0% | 3,531 | 6,917 | 2,750 |
| Control | CBTM-364B_LV_cells | 185,481,805 | 80.9% | 1,822 | 1,790 | 688 |
|  | CBTM-390C_LV_cells | 59,945,968 | 55.8% | 4,104 | 914 | 128 |
|  | CBTM-417C_LV_cells | 210,303,223 | 70.2% | 6,470 | 4,237 | 2,062 |
|  | CBTM-473C_LV_cells | 170,311,567 | 57.4% | 6,317 | 1,313 | 526 |

CBTM, Cambridge Biorepository for Translational Medicine; HFrEF, heart failure with reduced ejection fraction; LV, left ventricle; UMI, unique molecular identifier.

*Sequencing saturation equals the fraction of reads originating from an already-observed UMI (unique molecular identifier, equivalent to a unique detected transcript).
† median number of UMIs detected per cell.

‡ median number of genes detected per cell.

**Section D. Supplemental Data sets (Excel / CSV file format)**

For Table S3-S5, please refer to appended xlsx-files:

*Table S3. Top 50 Cluster and sub-cluster marker genes.xlsx*

*Table S4. Upregulated and downregulated genes in HFrEF.xlsx
 Table S5. Gene list classified in modules for trajectory analysis.xlsx*

For gene ontology enrichment analysis, please refer to appended csv-files:

*EGO_gene module 1-7 (csv)
GO.FAP.down.csv*

*GO.FAP.up.csv*
